# Artificial cell synthesis using biocatalytic polymerization-induced self-assembly

**DOI:** 10.1038/s41557-023-01391-y

**Published:** 2023-12-04

**Authors:** Andrea Belluati, Sètuhn Jimaja, Robert J. Chadwick, Christopher Glynn, Mohamed Chami, Dominic Happel, Chao Guo, Harald Kolmar, Nico Bruns

**Affiliations:** 1 Department of Pure and Applied Chemistry, University of Strathclyde, Thomas Graham Building, Glasgow, UK; 2 Department of Chemistry and Centre for Synthetic Biology, Technical University of Darmstadt, Darmstadt, Germany; 3 Adolphe Merkle Institute, University of Fribourg, Fribourg, Switzerland; 4 Biozentrum, University of Basel, Basel, Switzerland

**Keywords:** Molecular self-assembly, Polymer synthesis, Synthetic biology, Origin of life, Nanoscale materials

## Abstract

Artificial cells are biomimetic microstructures that mimic functions of natural cells, can be applied as building blocks for molecular systems engineering, and host synthetic biology pathways. Here we report enzymatically synthesized polymer-based artificial cells with the ability to express proteins. Artificial cells were synthesized using biocatalytic atom transfer radical polymerization-induced self-assembly, in which myoglobin synthesizes amphiphilic block co-polymers that self-assemble into structures such as micelles, worm-like micelles, polymersomes and giant unilamellar vesicles (GUVs). The GUVs encapsulate cargo during the polymerization, including enzymes, nanoparticles, microparticles, plasmids and cell lysate. The resulting artificial cells act as microreactors for enzymatic reactions and for osteoblast-inspired biomineralization. Moreover, they can express proteins such as a fluorescent protein and actin when fed with amino acids. Actin polymerizes in the vesicles and alters the artificial cells’ internal structure by creating internal compartments. Thus, biocatalytic atom transfer radical polymerization-induced self-assembly-derived GUVs can mimic bacteria as they are composed of a microscopic reaction compartment that contains genetic information for protein expression upon induction.

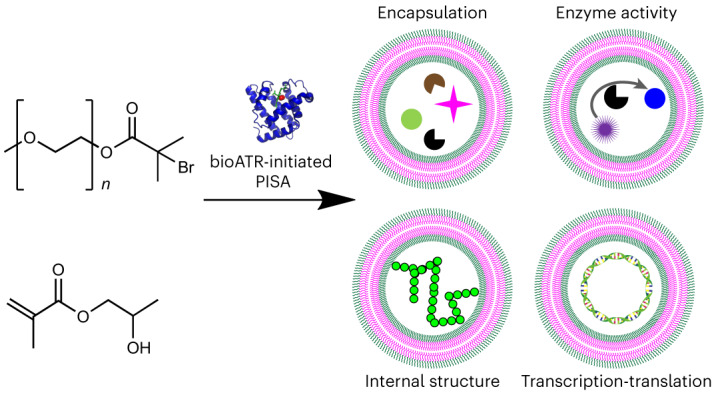

## Abstract (web summary)

Enzyme-initiated polymerization-induced self-assembly has been used to generate various biomimetic structures. Now, myoglobin’s activity is used for biocatalytic polymerization-induced self-assembly to generate vesicular artificial cells. As various cargoes can be encapsulated during polymerization, these artificial cells are capable of protein expression and can act as microreactors for distinct enzymatic reactions.

## Main

The ability to imitate life formation processes holds exciting potential for understanding and controlling biological functions and addressing questions about the origins of life^1–4^. Creating a fully artificial cell (also known as cell mimic) remains a remarkable challenge; however, researchers have made substantial progress in emulating various aspects of living systems, such as cell-like structures^5–7^, growth and replication^8,9^, communication^10,11^, responsiveness^12–14^, protein expression^12–14^ and enzymatic activity^13,15^. Moreover, artificial compartments have been previously used for cell-free transcription–translation (TX–TL) studies^16^. Biomimetic compartment structures are essential components of artificial cells and include polymersomes, liposomes, coacervates, colloidosomes, virus-like particles and protein cages^17–22^. In particular polymersomes have attracted a lot of attention because they have several advantages over non-polymer-based self-assemblies, including higher stability. Moreover, the chemical versatility of polymers can be leveraged to design polymersomes with multiple molecular functionalities such as degradability, stability, permeability and stimulus responsiveness^14,23–29^. Most bottom-up approaches to synthesize polymersomes have limitations due to the complexity of the preparation methodology or because of the low encapsulation efficiency (EE) of functional cargo.

To address these limitations, researchers have turned to polymerization-induced self-assembly (PISA) as an effective method to produce polymer-based biomimetic structures, including polymersomes^26,30,31^. PISA involves synthesizing amphiphilic block co-polymers in aqueous solution by polymerizing a water-soluble monomer into a water-insoluble polymer block, using a hydrophilic polymeric initiator or chain transfer agent for the hydrophilic block. During the initial phase of polymerization, the forming amphiphilic block co-polymer is still soluble in water. Once the hydrophobic block has reached a certain length, the co-polymers self-assemble. The hydrophobic block continues to grow in length so that the hydrophilic-to-hydrophobic ratio of the block co-polymer decreases, and as a result, the morphology of the self-assembled structures evolves from micelles, over worm-like micelles, into vesicles. PISA is usually conducted with reversible-deactivation radical polymerizations, most prominently with reversible addition-fragmentation chain-transfer polymerizations^26,31–34^, but also with atom transfer radical polymerizations (ATRP)^35^. PISA works under concentrated reagents conditions, resulting in high concentrations of self-assembled objects, and it allows highly efficient encapsulation of cargo in aqueous solutions in a one-pot procedure^26,32–34^. Recently, PISA has been employed in the synthesis of biomimetic structures such as giant unilamellar vesicles (GUVs) and polymersome nanoreactors, demonstrating the potential of this technique in the pursuit of artificial cells^24,31,36–39^.

**Fig. 1:**
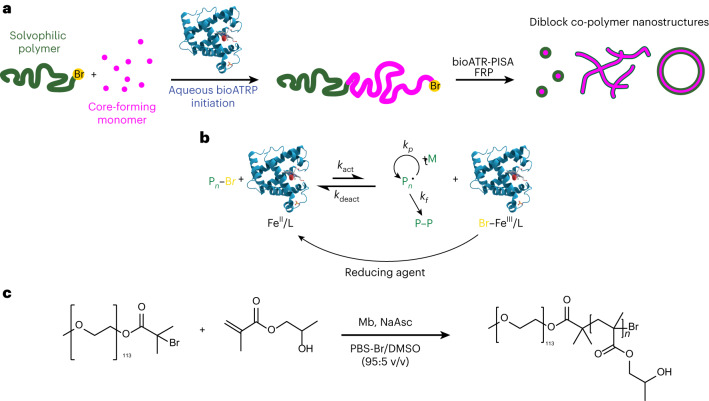
The bioPISA reaction of HPMA initiated by mPEG-Br to yield amphiphilic block co-polymers. **a**, Scheme of the bioPISA process that produces various self-assembled structures in aqueous solutions, using bioATRP that then evolves in free radical polymerization (FRP). **b**, Mechanism of Mb-mediated bioATRP. **c**, Reaction scheme of bioPISA by chain extension of a PEG-BiB macroinitiator with HPMA in aqueous solution resulting in the amphiphilic diblock co-polymer mPEG-*b*-PHPMA.

Incorporating biological molecules in artificial cells requires biocompatible conditions, with PISA holding an advantage as it occurs in aqueous solutions. However, initiation conditions can potentially harm sensitive biological molecules. For instance, the high temperatures required for thermal initiators are not ideal when working with biomolecules. Light-triggered polymerizations are safer, especially when visible light is used, but biologically derived initiation may be ideal. An elegant approach would be to use biocatalytic initiation of reversible-deactivation radical polymerization systems^40^. Recently, an enzyme-initiated PISA was reported by Tan and co-workers, which employed horseradish peroxidase to initiate reversible addition-fragmentation chain-transfer polymerization^41,42^. However, this approach necessitates hydrogen peroxide and the formation of free radicals to initiate the reaction, which could lead to cytotoxicity^43^. Moreover, the use of biologically relevant buffers as solvent led to unstable nanoparticles and as such de-ionized water was employed. Therefore, a more robust biocatalytic polymerization-induced self-assembly (bioPISA) technique is needed to couple the synthesis and self-assembly of polymers through PISA with biological or biomimetic systems. Biocatalytic ATRP (bioATRP) uses enzymes or their co-factors to initiate and control radical polymerizations and was introduced simultaneously by our group^40,44,45^ and di Lena and co-workers^46,47^. A wide range of biologically derived catalysts have been employed as ATRPase, such as horseradish peroxidase^44^, laccase^46^, catalase^47^, haemoglobin^45,48,49^ and myoglobin (Mb)^50^. This paves the way for a biocatalytic PISA methodology based on enzyme-mediated ATRP (Fig. 1a).

In this Article, connecting the worlds of biopolymerizations and life mimesis, we report the development of bioPISA and its application as a biocompatible methodology to synthesize synthetic cell-like structures. The scope of conditions for bioPISA was investigated, demonstrating its robustness in different media useful for biological applications. To achieve biomimetic systems, different enzymes were encapsulated in GUV synthesized by bioPISA, acting as microreactors, thus showing that the activity of the encapsulated enzymes was retained. Other proteins were instead used to trigger the formation of cytoskeleton-like structures and for osteoblast-inspired biomineralization. Moreover, as PISA achieves high EEs, it is well suited for the creation of complex cell mimics. Thus, bacterial cell lysate and different plasmids were encapsulated inside the GUVs, effectively reconstituting a functional cytoplasm with a simplified chromosome within an artificial cell membrane. The resulting artificial cells were capable of expressing several proteins, which were, in turn, used to alter the artificial cell’s internal structure.

## Results and discussion

### bioPISA

The bioPISA procedure was developed with biocompatibility and ease of use in mind. As such, we selected biocompatible chemicals according to the principles delineated by Matyjaszewski and co-workers^51^, and the reaction steps were simplified as much as possible. Mb was selected as biocatalyst because it is a small, robust and unimeric protein that functions as ATRPase at a biologically relevant pH of 7.4 (Fig. 1b), unlike haemoglobin, which requires acidic pH to become an ATRPase^45,48^. Moreover, Mb also displays peroxidase activity^52^, which is of relevance for the preparation of enzymatically active self-assemblies, as discussed below. In a typical bioPISA, the hydrophilic macromolecular ATRP initiator poly(ethylene glycol) methyl ether 2-bromoisobutyrate (mPEG-BiB) was chain-extended with 2-hydroxypropyl methacrylate (HPMA), which is the most commonly used monomer for PISA^26^. The reaction was carried out in a phosphate buffer solution at pH 7.4 with 100 mM of NaBr (PBS-Br) containing 5 v/v% dimethyl sulfoxide (DMSO), Mb and the reducing agent sodium ascorbate (NaAsc) at 37 °C (Fig. 1c). By adjusting the monomer:initiator ratio between 150 and 400, various degrees of polymerization (DP) were targeted (referred as aimed DP throughout the text, independently of the experimentally obtained values), and the monomer concentration was varied between 10 wt% and 25 wt%, (Supplementary Fig. 1). The reactions were run for 4 h before they were stopped by opening the reaction mixtures to air. The formation of nanostructures was easily recognized with the naked eye as after 1 h the reaction mixtures were already opaque (Supplementary Fig. 2). Proton nuclear magnetic resonance spectroscopy showed that after less than 2 h the monomer consumption usually plateaued, indicating the end of the reaction (Supplementary Fig. 3). Moreover, kinetic data displayed the characteristic shape for a PISA process with a hastening of the monomer consumption at the onset of aggregation (Supplementary Fig. 4)^33^. The Mb-mediated PISA proceeded faster than Mb-catalysed ATRP in solution, as PISA conditions compared with conventional solution polymerization often lead to high monomer consumption in shorter reaction time^53,54^. When a monomer concentrations of >10 wt% was used in the reactions, the final monomer consumption, except for high monomer:initiator ratio and lower wt% conditions, exceeded 90%, which indicated near complete conversion (Supplementary Table 1). In contrast, when a lower concentration of monomer was employed (5 wt%), a lower monomer consumption occurred (<60%). At the same time, the reaction for high aimed DPs (that is, lower initiator concentration) also yielded low monomer consumption, which established that a certain threshold of monomer and initiator concentration was necessary for the reaction to reach high yields. bioPISA from a small molecule initiator (2-hydroxyethyl 2-bromoisobutyrate) led to the formation of a macroscopic precipitation. This is certainly a consequence of the absence of mPEG as hydrophilic block in the forming polymers, which acts as stabilizing hydrophilic corona for the forming self-assemblies.

**Fig. 2:**
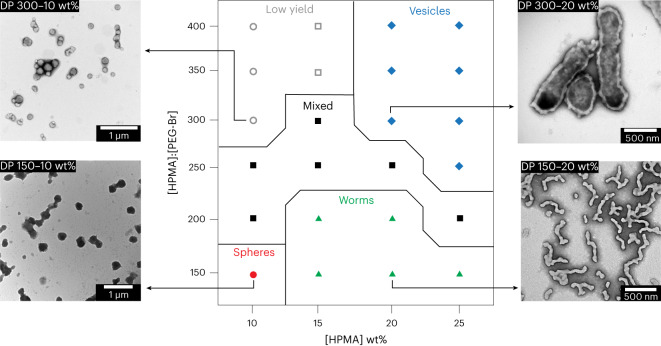
Structures derived from bioPISA. The phase diagram of bioPISA from a PEG macroinitiator with varying content of HPMA (wt%) and [HPMA]:[PEG-Br] ratio (that is aimed DP) of HPMA with illustrating dry state TEM images of the different morphologies. Morphology: sphere (circle), worms (triangle), vesicles (diamond) and mixture (square). Monomer consumption: ≥85% (full symbols) and <85% (empty symbols).

One of the key features of PISA is that it allows one to tailor the morphology of the resulting structures by varying parameters such as the monomer:initiator ratio or the monomer concentration in the reaction mixture^26,31,32^. The case of bioPISA is no different, as the aimed DP of polyHPMA and the concentration of monomer had a clear effect on the morphology, as analysed by transmission electron microscopy (TEM) and dynamic light scattering (DLS). These characterizations allowed us to establish a phase diagram representing the morphology of the self-assembled structures as a function of the monomer:initiator ratio and the initial monomer concentration (Fig. 2 and Supplementary Figs. 5–8). Spherical objects with an average diameter of 227 nm, as determined from TEM, were obtained at aimed DP 150 and 10 wt%. This size was confirmed by DLS analysis with a hydrodynamic diameter (*D*_h_) of 238 ± 22 nm (Supplementary Fig. 9). Worm-like micelles were obtained at aimed DP 150 and 200 with ≥15 wt% and 15–20 wt% of monomer, respectively. The worm-like micelles had an average diameter of 73 ± 15 nm as measured by TEM. Interestingly and in accordance with what has been reported before for worm-like micelles synthesized by PISA, the reaction mixtures containing worm morphologies thickened and became self-standing gels (Supplementary Figs. 6–8)^55^. Increasing the monomer:initiator ratio between 200 and 300, we could observe an intermediate region of mixed structures, between spheres, worms, and proper vesicles (polymersomes), as longer polymers could self-assemble into diverse shapes; however, at even monomer:initiator ratio and lower wt% (above 300 and below 20, respectively), the polymerization yield was <60%, with either mixed or spherical morphologies. Vesicles (polymersomes) were obtained with monomer:initiator ratio of 250 to 400 at monomer concentrations of ≥20 wt%. The diameter of the polymersomes ranged from a few hundred nanometres up to several micrometres, as imaged by TEM and confocal laser scanning microscopy (CLSM) (Supplementary Fig. 10). This broad range of sizes meant that it was not possible to fully analyse the suspensions by light scattering. The membrane of the polymersomes is clearly visible in the TEM images with a thickness of 50 ± 11 nm. The micrometre-sized polymersomes are GUVs and therefore ideally suited as shell of an artificial cell.

All three morphologies were further analysed by cryo-TEM. Similar structures to those imaged by dry-state TEM were found (Supplementary Fig. 11). However, the spherical objects showed internal structures resembling spherically entangled worm-like micelles (20% of the vesicles presented internal structures, *n* = 74), similar to those reported by Lee et al. for mixtures of co-polymers with high- and low-molecular-weight hydrophobic blocks^56^. The polymersome membrane can also be clearly identified in the cryo-TEM images, and an average thickness of 44 ± 10 nm was measured.

The molecular weight and therefore the DP of the obtained polymers could not be accurately measured, as substantial parts of the formed polymers were insoluble in common solvents, most likely because some of the poly(HPMA) blocks crosslinked. This phenomenon is regularly observed in PISA with HPMA^57,58^.

For the soluble polymer fraction, the measured DP was decoupled from the aimed DP (see discussion in Supplementary Information and Supplementary Fig. 12), which probably indicates that the reaction initiates as ATRP, but upon self-assembly of the chains, it transitions to proceed via free radical polymerization. Nevertheless, the morphology of the self-assembled structures could be reliably and reproducibly targeted through the monomer:initiator ratio and the monomer concentration, as evidenced by the multiple polymersome batches synthesized for the various experiments of reported in this work.

To interface bioPISA with biological systems it is important that bioPISA can not only be carried out in simple buffers, but also in complex biological media. To this end, bioPISA was conducted in three different cell culture media: Luria–Bertani, Minimum Essential Medium and RPMI-1640, aiming for spheres, worms and vesicles. The reactions yielded high monomer consumption along with self-assemblies of the same morphology as the reactions in PBS-Br (Supplementary Figs. 9 and 12–14, and Supplementary Table 2). However, vesicles appeared slightly larger, especially in RPMI, suggesting that the compounds in the reaction mixture might affect the self-assembly, a long-known phenomenon^59^. Moreover, when bioPISA was carried out in the presence of *Escherichia coli*, the bacteria remained viable, although it resulted in large microparticles rather than proper vesicles (Supplementary Figs. 15 and 16). Taken together, these results prove the robustness of bioPISA to synthesize structures in biologically relevant aqueous solutions and open the road to an easy, enzyme-catalysed synthesis of self-assemblies for biomaterials, living matter encapsulation or in situ formation of biomimetic nano-objects and synthetic cells. As GUVs are the required morphology for artificial cells, all further bioPISA experiments were carried out under conditions targeting a DP of 400 and with a monomer concentration of 25 wt% (Supplementary Table 2).

### Encapsulation by bioPISA

**Fig. 3:**
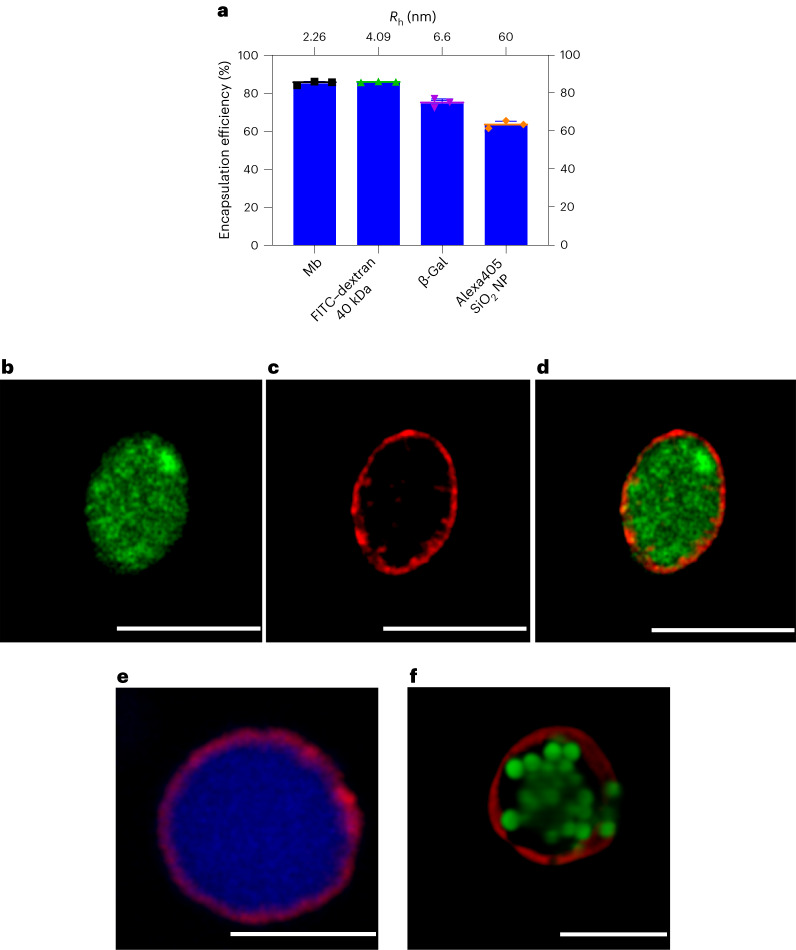
Encapsulation of biomacromolecules, nanoparticles and microparticles into polymersomes by bioPISA. **a**, EE for enzymes, dextran and silica nanoparticles by bioPISA as measured by UV–vis and fluorimetry (values as mean ± s.d., *n* = 3 replicates; *R*_h_ measured via DLS for the SiO_2_ NPs, obtained from the literature for other species). **b**–**d**, CLSM micrographs of a FITC–dextran 40 kDa-loaded GUV, showing the successful encapsulation: FITC–dextran 40 kDa (**b**), Cy5–PEG_3.5k_–cholesterol membrane stain (**c**), and overlay (**d**). Scale bar, 5 µm. **e**, Overlay CLSM micrograph of Alexa405 SiO_2_NP-loaded GUVs, with the NPs clearly encapsulated (blue: NPs, red: Cy5–PEG_3.5k_–cholesterol). Scale bar, 5 µm. **f**, CLSM 3D section of a GUV harbouring PS-μP FITC, with microparticles clumping together inside of the GUV (green: microparticles, red: Cy5–PEG_3.5k_–cholesterol). Scale bar, 10 µm. General reaction conditions: [HPMA] 20 wt%, aimed DP: 400. Error bars as mean ± s.d., *n* = 3. Source data

The first step towards the creation of artificial cells with bioPISA-derived micro-sized vesicles is to mimic the ability of cells to encapsulate macromolecules^60,61^, particles and internal compartments^62^ to create a complex environment in the lumen of the GUVs. To show that bioPISA vesicles can encapsulate compounds with a range of different sizes, bioPISA reactions were run in the presence of 40 kDa FITC-labelled dextran, β-galactosidase (β-gal; 102 kDa), and fluorescent silica nanoparticles (Alexa405 SiO_2_ NP). The EE of these cargoes, along with Mb that self-encapsulates, was ~85% for Mb, and decreased with the increase of the species’ *R*_h_ to 60% for the silica nanoparticles (Fig. 3a). CLSM imaging, which is limited by its resolution to the GUVs present in the mixture, showed that each of the fluorescent species was encapsulated into the GUVs (Fig. 3b–e and Supplementary Fig. 17). Even fluorescent polystyrene microparticles (PS-μP FITC, Fig. 3f) could be encapsulated, but they tended to clump together during bioPISA, some vesicles to be empty, and some to contain several particles. It should be noted that the GUV membrane was confirmed to be impermeable for macromolecules (Supplementary Figs. 18 and 19), so that the GUVs would not lose their cargo once the vesicles have formed in bioPISA. Size and shape of the GUVs were analysed. The majority of vesicles were elongated, that is had an aspect ratio <1, with a weak negative correlation between aspect ratio and size regardless of the macromolecular cargo (Supplementary Fig. 20a–c). However, the encapsulation of Alexa405 SiO_2_ NP slightly shifted the values towards smaller and rounder GUVs, which is an indication that the NP influence the self-assembly (Supplementary Fig. 20d). Monomer consumption remained high regardless of the cargo in the reaction mixture, including bacteria (Supplementary Fig. 21).

### bioPISA GUVs as microreactors for encapsulated enzymes

**Fig. 4:**
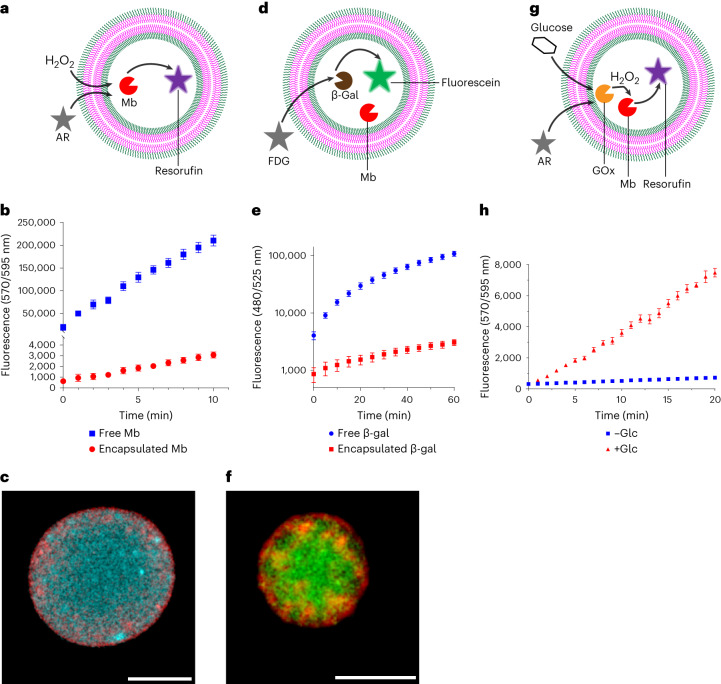
bioPISA-derived GUVs as microreactors for enzymes. **a**, Scheme of a one-enzyme microreactor, producing fluorescence from external substrates. **b**, Peroxidase activity of encapsulated and free Mb with H_2_O_2_ and AR. **c**, CLSM micrograph of a Mb-containing GUV after the reaction (cyan: resorufin, red: Cy5–PEG_3.5k_–cholesterol). **d**) Scheme of an artificial cell with two co-encapsulated enzymes, producing fluorescence from external substrates. **e**, Hydrolase activity of encapsulated and free β-gal with FDG. **f**, CLSM micrograph of a β-gal + Mb-containing GUV after the reaction (green: fluorescein, red: Cy5–PEG_3.5k_–cholesterol). **g**, Scheme of an artificial cell with two co-encapsulated enzymes producing fluorescence from external substrates following a cascade reaction. **h**, Peroxidase activity of encapsulated GOx + Mb cascade with AR. All values displayed as mean ± s.d., *n* = 3 replicates for all experiments. Scale bars for all images, 5 µm. Source data

The second fundamental feature for a cell-like system is the presence of a metabolism, that is, the ability to perform localized chemical reactions, as both real and synthetic cells are effectively microreactors^63,64^. This generally requires catalysts, where enzymes play this role in biological settings, able to conduct the required reactions in the cells. Even without the encapsulation of an additional enzyme, the bioPISA-derived GUVs are microreactors themselves as they host Mb that self-encapsulates during the PISA process, and can be imaged in the GUVs once properly labelled (Supplementary Fig. 22a). The enzyme was distributed throughout the lumen of the GUVs, that is it did not accumulate on or at the polymer wall of the vesicles. The capability of encapsulated Mb to transform the substrate Amplex Red (AR) into the fluorescent resorufin in the presence of H_2_O_2_ was monitored (Fig. 4a). Fluorescence increased over time (Fig. 4b) and was initially detectable only within the lumen of GUVs, until resorufin eventually diffused out (Fig. 4c, Supplementary Fig. 23a and Supplementary Video 1). Thus, the Mb was active inside of the polymersomes and the polymersome membrane was permeable for substrates and products. These observations are in line with previous studies in which PISA-derived mPEG-*b*-PHPMA vesicle membranes were shown to be permeable for small water-soluble compounds, but impermeable for macromolecules^15^. At the same enzyme concentration, encapsulated Mb exhibited a lower activity than free Mb (246 relative fluorescence units (RFU) min^−1^ versus 18,000 RFU min^−1^) (Fig. 4b). The decrease in specific activity might be a consequence of the slow diffusion of the substrate across the membrane^65^, although it cannot be ruled out that some of the enzyme is irreversibly deactivated. Indeed, Mb that was incubated in the reaction mixture of bioPISA without any polymerization retained only 21% of its activity (Supplementary Fig. 24), indicating that the high concentration of monomer and other reagents during bioPISA led to a partial deactivation of the enzyme.

The next step for the bioPISA GUVs towards artificial cells was to co-encapsulate enzymes acting in parallel to Mb but with an orthogonal activity (Fig. 4d and Supplementary Figs. 22b and 23b). β-Gal is a robust, widely used enzyme for industrial biotransformations and prodrug activation^66,67^, and was an ideal candidate as it does not interfere with Mb activity. Similarly to Mb, β-gal activity (the hydrolysis of fluorescein di-β-d-galactopyranoside, FDG) was lower in the vesicles than for free enzyme (36 RFU min^−1^ versus 1,730 RFU min^−1^) (Fig. 4e), and the resulting fluorescein concentrated within the lumen of the GUVs (Fig. 4f). The lower activity within the vesicles could be due to due to diffusion limitations by the membrane and deactivation of the enzyme during bioPISA. When exposed to the polymerization reaction mixture, β-gal also retained only 40% of its activity (Supplementary Fig. 24). Thus, the bioPISA reaction conditions, while relying on the comparably mild ATRP, do affect the enzymes, similar to previously reported enzyme encapsulation by conventional PISA^15,24,68^. Finally, a glucose oxidase (GOx)–Mb enzyme cascade^69^ was established in the GUVs where the formation of the final product requires the synergy of two co-encapsulated enzymes (Fig. 4g). Driven by the GOx-catalysed reaction of glucose and oxygen into glucono-1,5-lactone and H_2_O_2_, followed by a peroxidase reaction catalysed by Mb, the cascade led to the production of resorufin in the bioPISA-derived GUVs (Fig. 4h). Thus, the bioPISA GUVs served as microreactors for an enzyme cascade.

### Triggered structure modifications in bioPISA GUVs

A third characteristic of cells is their ability to respond to external stimuli, for instance by altering their shape and structure^70^. Several kinds of stimuli-responsive artificial cells have been designed in the past^71,72^, including polymer-based structures that change their shape upon a specific external trigger^73–76^, but so far none can compete with actual cells in complexity.

**Fig. 5:**
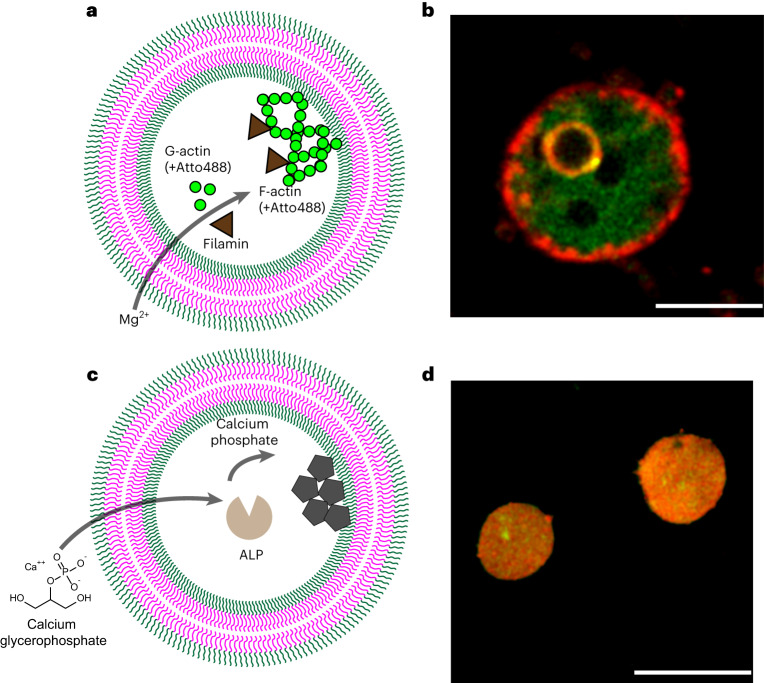
Triggered structure modifications in bioPISA GUVs. **a**, Scheme of the Mg^2+^-induced polymerization/condensation of actin in GUVs. **b**, CLSM micrograph of a GUV after the formation of actin condensates (actin/actin–Atto488/filamin: 1/0.25/0.01 weight ratio; green: actin–Atto488, red: Cy5–PEG_3.5k_–cholesterol; the cholesterol dye could only label some of the inner compartments). Scale bar, 5 µm. **c**, Scheme of ALP-mediated calcium phosphate precipitation within GUVs. **d**, CLSM micrograph of a GUV after the biomineralization of CaGP by ALP, having become a polymer-mineral capsule. Green: calcium phosphate-adsorbed fluorescein. Red: Cy5–PEG_3.5k_–cholesterol. Scale bar, 10 µm.

An example of such structural complexity is the formation of cytoskeleton-like structures. The cytoskeleton component actin is known to produce tight, stiff networks together with the crosslinker filamin, once it polymerizes from G-actin to F-actin at high Mg^2+^ concentrations^77–79^. Using bioPISA to encapsulate filamin and fluorescently labelled actin, we observed magnesium-triggered changes in GUVs’ internal architecture (Fig. 5a). This led to subcompartmentalized vesicles with condensed actin, resembling a hybrid of poly(dimethylsiloxane)-poly-b-(methyloxazoline) (PDMS-PMOXA) GUVs encapsulating actin (which can form internal vesicles)^77^, and actinosomes (which create protein shells around coacervates)^80,81^ (Fig. 5b and Supplementary Fig. 25). These structures did not form in absence of Mg^2+^ (Supplementary Fig. 26) or without actin (Supplementary Fig. 27), confirming that actin polymerization in the confined space of the GUVs let to their formation. The relationship between area, aspect ratio and filling ratio of each vesicle shows that the actin polymerization does not modify the shape or size of GUVs, but clearly induces a decrease in the overall filling ratio (Supplementary Fig. 28), an indicator of the GUV compactness.

Many cell types do not rely only on biomolecules to modify their architecture, but can also use minerals to produce matrices and scaffolds^82^, becoming of interest for materials science and architecture as well^83^. A biomineralizing system was developed, where encapsulated alkaline phosphatase (ALP) (Supplementary Fig. 29) converted the dissolved calcium glycerophosphate (CaGP) to insoluble calcium phosphate^84^. This precipitation reaction imitated the biological mechanism in which osteoblasts produce the mineral matrix of bones (Fig. 5c). The reaction produced the expected white precipitate of calcium phosphate only in presence of ALP in the GUV and CaGP (Supplementary Fig. 30) added outside. By adsorbing fluorescein to the mineral^85^, the resulting particles could be imaged (Fig. 5d and Supplementary Fig. 31). Due to the intravesicular localization of ALP, the calcium phosphate precipitated within the GUVs, causing the destabilization of the polymer membrane, which adsorbed on the phosphate particles. Thanks to an encapsulated enzyme, the GUVs could turn into a new organic–inorganic hybrid material, where the biomineral could be wholly contained within the polymer matrix.

### GUVs as bacteria mimics capable of protein expression

**Fig. 6:**
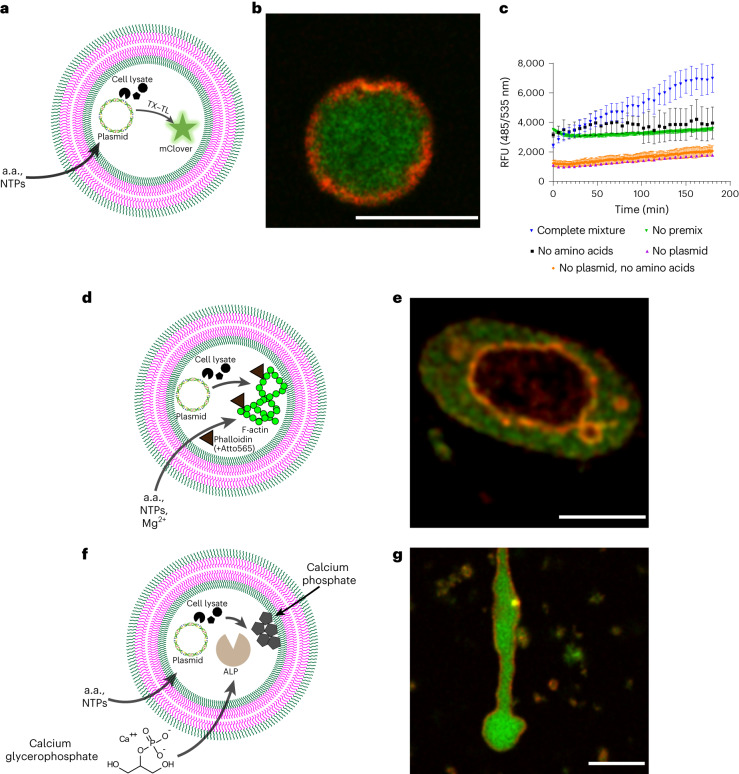
bioPISA GUVs as artificial bacterium capable of protein expression. **a**, Scheme of cell-free expression of mClover in GUVs, where amino acids (a.a.) and NTPs are provided from the outside to a GUV encapsulating the plasmid and the S30 cell lysate, resulting in the expression of the fluorescent protein. **b**, CLSM micrograph of a GUV after expression of mClover. Green: mClover. Red: Cy5–PEG_3.5k_–cholesterol. Scale bar, 10 µm. **c**, Expression profiles of the fluorescent mClover in GUVs with no plasmid (baseline), plasmid alone or plasmid and amino acids. All values displayed as mean ± s.d., *n* = 3 replicates for all experiments. **d**, Scheme of a GUV containing a plasmid and bacterial translation machinery, producing an actin cytoskeleton. Scale bar, 10 µm. **e**, CLSM micrograph of a GUV with expressed actin + phalloidin-Atto565 (green: phalloidin-Atto565, red: Cy5–PEG_3.5k_–cholesterol). Scale bar, 10 µm. **f**, Scheme of a GUV containing a plasmid and bacterial translation machinery for the production of ALP, and subsequent ALP-catalysed biomineralization of calcium phosphate. **g**, CLSM micrograph of a GUV after the biomineralization of CaGP by expressed ALP. Green: calcium phosphate-adsorbed fluorescein. Red: Cy5–PEG_3.5k_–cholesterol. Scale bar, 10 µm. Source data

A key feature of cells is their ability to express proteins through the DNA transcription and RNA translation (TX–TL mechanism). Thus, encapsulating a cell-free expression system, that is, cell cytoplasm into bioPISA GUVs could potentially reconstitute this cellular function and turn them into artificial cells. We developed a reaction mixture for bioPISA that contained *E. coli* lysate (S30 cell free protein expression system, containing 25% of the total bacterial proteome^86^) and a plasmid of interest, so that all proteins, ribosomes, transfer RNAs and the plasmid were encapsulated into the GUVs. Amino acids were added from the outside to the GUVs, as well as the Premix, containing nucleotides (NTPs), salts, isopropyl β-d-1-thiogalactopyranoside and pyruvate kinase for ATP regeneration^16,87^. The TX–TL capability of this system was confirmed with the expression of the fluorescent mClover protein in the bioPISA GUVs (Fig. 6a), yielding fluorescent vesicles as confirmed by confocal microscopy (Fig. 6b and Supplementary Fig. 32a). The semiquantitative analysis of the fluorescence signal over time (Fig. 6c) shows a quick baseline increase once the reaction is started, but the protein expression can only be sustained on the longer term when both amino acids and the Premix are added to plasmid-containing GUVs, and otherwise can only rely on residual molecules (amino acids and NTPs) in the cell lysate^88,89^. Analysis by CLSM (Supplementary Fig. 32b) shows that higher fluorescence intensities were detected in smaller GUVs, suggesting that the protein is diluted within the volume of larger vesicles. Flow cytometry confirmed the different fluorescence intensities with and without mClover expression (Supplementary Fig. 33). These results clearly show that bioPISA resulted in bacteria-mimetic structures, that is, microscopic reaction compartments with a single ‘chromosome’ (the plasmid) with no subcompartmentalization, capable of expressing proteins upon induction. Fascinatingly, this expression could be repeated 1 year later with GUVs stored at 4 °C, which maintained 64 ± 16% of their protein expression activity (Supplementary Fig. 34). This result was not fully unexpected, as polymer encapsulation is known to stabilize biological components^23^.

Cells are also able to modify their internal structure, and need to express specific proteins to that end. As we demonstrated above, the condensation of F-actin produced internal compartments in the GUVs. Intrigued by this phenomenon, actin was expressed in the presence of fluorescently labelled phalloidin (Fig. 5d), to promote the polymerization of actin even at low concentrations^90^ and allow its visualization. At the same conditions of the pre-encapsulated actin, we could observe actin condensation leading to internal compartments (Fig. 5e and Supplementary Figs. 35 and 36), suggesting once again that actin polymerization induced structural changes in the GUV.

Similarly, ALP was expressed as functional enzyme (Fig. 6f,g and Supplementary Fig. 37) and induced calcium phosphate precipitation (Supplementary Fig. 38). In contrast to the studies above, the enzyme formed calcium phosphate cores inside of the GUVs that in some cases extended and deformed the vesicle membrane so that protrusions formed (Fig. 6f and Supplementary Fig. 39). We speculate that the difference in morphology between GUV-calcium phosphate structures obtained by ALP expressed in situ compared with directly encapsulated ALP might be a consequence of a lower concentration of enzyme inside of the GUVs in the former case, causing a lower production of calcium phosphate that then precipitated within the lumen of the vesicles without destabilizing their membranes.

The three examples of protein expression within bioPISA-derived GUVs show that polymer-based artificial cells could be produced by enzymatic polymerization while simultaneously encapsulating the TX–TL machinery that expressed proteins and enzyme that in turn modified the structure of the artificial cells. Thus, these ‘artificial bacteria’ possessed emergent structural properties.

## Conclusions

In this work, we have demonstrated how an enzymatic reaction can induce the formation of polymeric vesicles with increasing levels of functional complexity, from simple microcapsules to microreactors and complex cell mimics that are capable of producing their own proteins, thanks to co-encapsulated plasmids as genetic information. An enzyme could self-encapsulate within a compartment formed by a non-natural membrane that the enzyme synthesized itself, entrapping all the complex components deriving from cell lysates, which in turn could express specific proteins and modify the structure of the vesicles, producing internal compartments, or transforming them into polymer-mineral hybrids. Cell mimics were produced in the most biosimilar process possible, showcasing the versatility and robustness of bioCRP. Moreover, bioPISA proved to be a very efficient and biocompatible encapsulation method for a range of cargo. Although the bioPISA-derived GUVs are still simplified mimics of actual cells, bioPISA will pave the way for hybrid systems combining synthetic polymers with the diversity of natural molecules, offering a plethora of applications in synthetic biology, sensing, catalysis and biomaterials.

## Methods

### bioPISA

In a typical bioPISA experiment (scheme 2), HPMA (2 ml, filtered on basic alumina) was measured in a 4 ml vial that was closed with a septum. A certain amount of mPEG-BiB and NaAsc was dissolved in a certain volume PBS-Br buffer pH 7.4 spiked with 5 vol% DMSO in a 4 ml vial closed with a septum. A certain amount of Mb was suspended in PBS-Br buffer pH 7.4 in a 10 ml Schlenk flask. All the solutions were degassed with Ar for 1 h. The mPEG-BiB/NaAsc solution was added to the Schlenk flask, and the resulting solution was stirred for 15 min. The colour of the reaction mixture changed from brown to red (Mb oxidation). Then, purified HPMA was added. The reaction mixture was stirred for 4 h at room temperature before being opened to air to quench the polymerization by atmospheric oxygen. The final suspension was diluted with 15 ml of PBS. The reagent amounts are specified in Supplementary Table 1. All encapsulations were performed with HPMA 20 wt% and aimed DP 400.

### CLSM

Imaging of vesicles was performed on a Leica SP8 CLSM, equipped with an HCX PL APO 63× NA 1.2 W CORR CS2 objective (Alexa405: excitation (ex.) 405 nm, emission (em.) 410–430 nm; fluorescein, mClover, FITC, Atto488: ex. 488 nm, em. 505–525 nm; resorufin, Atto565: ex. 561 nm, em. 570–590 nm; Cy5, PI: ex. 635 nm, em. 660–690 nm). Membranes were stained with Cy5–PEG_3.5k_–cholesterol, synthesized according to a published procedure^91^. Images captured with the software LAS X v5.0 and were optimized (brightness and contrast; applied evenly throughout a whole image) and analysed via FIJI v1.53 (ref. ^92^).

### EE

Several cargoes were co-encapsulated together with Mb. Depending on the sample, we added to the reaction mixture:

- 32 µl FITC–dextran 40 kDa 10 mg ml^−1^
- 10 µl β-gal 10 mg ml^−1^
- 10 µl Alexa405 SiO_2_ NP 1% w/v%

To quantify the EE, post-polymerization aliquots were diluted 1:10 in PBS, and run through PD 10 desalting columns (Cytiva; gravity protocol) to remove the unencapsulated molecules. The second fraction (7 ml, per supplier’s instructions) was recovered as well. Alexa405 SiO_2_ NP were instead recovered by centrifuging a 1 ml aliquot of vesicles (500*g*, 3 min, in an Eppendorf microcentrifuge), and recovering the supernatant.

For FITC–dextran and Alexa405 SiO_2_ NP, a calibration curve was used to quantify the encapsulated fluorophores in the second fraction, on a Clariostar Plus plate reader (BMG Labtech), in a Greiner transparent 96-well plate, with the software ClarioStar Plus v6.5.

In the case of Mb, the absorbance at 280 nm in the second fraction was quantified, with *ε* = 13.98 mM^−1^ cm^−1^, from its sequence, as of (*N* of tryptophan residues × 5.5) + (*N* of tyrosine residues × 1.5) (ref. ^93^). For β-gal, the protein absorbance at 280 nm was likewise calculated, using the expected content of Mb as blank, with *ε* = 191.95 mM^−1^ cm^−1^. The measurements were conducted in a Cary 60 ultraviolet–visible (UV–vis) spectrophotometer (Agilent).

FITC-labelled polystyrene microbeads (2 µm diameter) were added to the reaction mixture (10 µl directly from the suspension) and then directly imaged via CLSM.

To quantify unencapsulated Mb after washing of the first fraction, the absorbance values after bicinchoninic acid assay (Merck Millipore, protocol according to manufacturer’s manual) were compared in GUVs, GUVs after centrifugation (500*g*, 3 min, in an Eppendorf microcentrifuge, 2×) and washing with PBS, and the resulting washing supernatant. Volumes of both GUVs and supernatant were kept constant.

### Enzyme activity of Mb

The enzymatic activity of Mb was measured in a Clariostar Plus plate reader, in a Greiner transparent 96-well plate. In each well, 10 µl of vesicles (with Mb alone, or with GOx co-encapsulated) were mixed with 2 µl Ampliflu Red (also known as Amplex Red, AR) 100 µM, 5 µl H_2_O_2_ 0.01%, to a final volume of 200 µl of PBS or PBS + 20 mg ml^−1^ glucose (with GOx present), and the fluorescence at 570/595 nm (±10 nm) was recorded, between 10 and 20 min.

### Enzyme activity of β-gal

To obtain β-gal-loaded vesicles, 100 µl of a 10 mg ml^−1^ enzyme solution was added to the reaction mixture. The enzymatic activity of β-gal was measured in a Clariostar Plus plate reader, in a Greiner transparent 96-well plate. In each well, 10 µl of vesicles encapsulating β-gal were mixed with 10 µl FDG, and the formation of fluorescein was followed at 495/535 (±10) nm.

### Actin polymerization

Actin was labelled with Atto488-NHS ester, by reacting 1 mg of protein in 1 ml PBS, with 1 mM dye, 37 °C, 1 h, and then purifying the mixture with a 10 kDa spin diafiltration device. Then 100 µl of actin 1 mg ml^−1^ and 10 µl of actin–Atto488 were added to the Mb mixture. The solvent used was Tris–HCl, pH 7.5, with the addition of 100 mM NaBr. After purification, the vesicles were imaged by diluting them either in Tris–HCl, or MgCl_2_ 100 mM.

### Calcium phosphate biomineralization

Ten microlitres of ALP (13 mg ml^−1^) were added to the reaction mixture. In an Eppendorf tube, 100 µl of the purified sample were mixed with 50 µl CaGP 100 mM and 5 µl fluorescein 10 mM, then incubated at 37 °C, 4 h. The cleaving of glycerophosphate (CaGP) by ALP was confirmed visually by the formation of a white precipitate. The samples were then imaged via CLSM.

### Plasmid encapsulation and cell-free expression

The cell-free expression of proteins within GUVs was achieved using the Promega *E. coli* S30 Extract Protein Expression System for Circular DNA (L1020). Seventy-five microlitres of plasmid, at different concentrations (pNCS–mClover3: 300 ng ml^−1^; ZP9A actin: 98.4 ng ml^−1^; Chx10 3 kb AP: 137 ng ml^−1^), and 50 µl of S30 EXTRACT (cell lysate) were added to the reaction mixture. For ZP9A actin, 10 µl of Phalloidin Atto565 (20 µM in MeOH) were added as well. After polymerization and purification via SEC in HEPES pH 8.5, to 70 µl of sample, 50 µl of S30 premix (containing the energy regenerating system, at pH 8.4) and 10 µl of complete amino acid mixture were added. The sample was incubated at 37 °C for 3 h, either split in three wells per condition (mClover synthesis) or in Eppendorf tubes. Afterwards, they were imaged via CLSM. Actin-containing vesicles were resuspended in 100 mM MgCl_2_, whereas ALP-containing ones were first incubated in CaGP according to the protocol described above.

### Reporting summary

Further information on research design is available in the Nature Portfolio Reporting Summary linked to this article.

## Online content

Any methods, additional references, Nature Portfolio reporting summaries, source data, extended data, supplementary information, acknowledgements, peer review information; details of author contributions and competing interests; and statements of data and code availability are available at 10.1038/s41557-023-01391-y.

### Supplementary information

Supplementary Information Expanded materials and methods, supplementary discussion, and Supplementary Tables 1 and 2 and Figs. 1–39.

Reporting Summary

Supplementary Video 1 Production of resorufin within GUVs.

### Source data

Source Data Fig. 3 Numerical data for Fig. 3.

Source Data Fig. 4 Numerical data for Fig. 4.

Source Data Fig. 6 Numerical data for Fig. 6.

## Data Availability

The datasets generated and analysed during the current study are available on Zenodo at 10.5281/zenodo.8414850. Numerical source data are provided for the graphs present in the manuscript. Source data are provided with this paper.
