# Supplementary material for: Artificial cell synthesis using biocatalytic polymerization-induced self-assembly
Source: Nat Chem. 2023 Dec 4;16(4):564–74. doi: 10.1038/s41557-023-01391-y (PMC10997521; doi:10.1038/s41557-023-01391-y)
Supplement: Supplementary file 1 — Expanded materials and methods, supplementary discussion, and Supplementary Tables 1 and 2 and Figs. 1–39. [file 41557_2023_1391_MOESM1_ESM.pdf]

nature chemistry

Article

https://doi.org/10.1038/s41557-023-01391-y

Artificial cell synthesis using biocatalytic polymerization-induced self-assembly

In the format provided by the authors and unedited

Table of Contents

Materials 2
Compounds 2
Plasmids 2
Experimental procedures 2
Synthesis of poly(ethylene glycol) methyl ether 2-bromoisobutyrate (mPEG-BiB) 2
BioPISA 3
COMPLETE TECHNIQUES 3
NMR spectroscopy 3
Size exclusion chromatography 3
Dynamic light scattering (DLS) 3
Reaction kinetics 4
Co-encapsulation of glucose oxidase (GOX) 4
Enzyme activity of alkaline phosphatase (ALP) 4
Polymerization in presence of bacteria 5
Flow cytometry 5
Transmission electronic microscopy 5
Cryo-transmission electronic microscopy 5
Morphological characterization of GUVs 5
Data analysis 6
SUPPORTING INFORMATION 7
Reaction conditions 8
Size exclusion chromatography 13
Biocompatibility and bacterial encapsulation 17
Reaction overview 18
Encapsulation and membrane permeability 20
Purification of GUVs via size-exclusion chromatography 20
Actin polymerization 24

MATERIALS

Compounds

Polyethylene glycol monomethyl ether (mPEG, 5 kDa), bromoisobutyryl bromide, triethylamine (Et_3N), sodium ascorbate (NaAsc), myoglobin (Mb), hydroxypropyl methacrylate (HPMA), Tris-HCl, dimethylformamide (DMF), 4-(dimethylamino)pyridine (≥98%, DMAP), RPMi-1640 (with addition of fetal bovine serum (10%), L-glutamine (1%), penicillin-streptomycin (1%)), FITC-dextran 40kDa, β-galactosidase (β-Gal), glucose oxidase (GOX), alkaline phosphatase (ALP), actin, Atto488 NHS-ester, phalloidin Atto565, calcium glycerophosphate (CaGP), fluorescein di(β-D-galactopyranoside) (FDG), fluorescein diacetate (FDA), and propidium iodide (PI) were purchased from Sigma-Aldrich and used as received. Tetrahydrofurane (THF) and dichloromethane (CH_2Cl_2) were purchased from Acros Organics. *E. coli* T7 S30 Extract Protein Expression System for Circular DNA was purchased from Promega. All solvents were purchased in high purity. All reactions requiring anhydrous conditions were carried out using dried Schlenk glassware and under inert Ar gas atmosphere. Deuterated solvents were purchased from Cambridge Isotope Laboratories. PBS 10X was prepared and NaBr was added to make PBS-Br 1X (NaBr 100 mM). Alexa405 SiO_2 NPs were a gift by Prof. Alke Petri-Fink (Adolphe Merkle Institute, University of Fribourg, Switzerland).[1]

Plasmids

pNCS-mClover3 was a gift from Prof. Markus Biesalski (TU Darmstadt).[2] ZP9 Actin was a gift from Randall Moon (Addgene plasmid # 16932; http://n2t.net/addgene:16932; RRID:Addgene_16932), Chx10 3kb AP (CC#318) was a gift from Connie Cepko (Addgene plasmid # 15205; http://n2t.net/addgene:15205; RRID:Addgene_15205).[3] pNCS-mClover3 was propagated in *E. coli* BL21 (DE3), whereas ZP9A and CHx10 3kbAP were propagated in *E. coli* DH5alpha. All cultures were grown in Luria-Bertani broth supplemented with the antibiotic they would be resistant to (ampicillin or kanamycin) until OD_{600} 1. Then, the plasmids were purified using a QIAprep Spin Miniprep Kit (Qiagen, USA). The amount of purified DNA was quantified at 260 nm using a NanoDrop One/One^C UV-Vis Spectrophotometer (Thermo Scientific).

EXPERIMENTAL PROCEDURES

SYNTHESIS OF POLY(ETHYLENE GLYCOL) METHYL ETHER 2-BROMOISOBUTYRATE (MPEG-BIB)

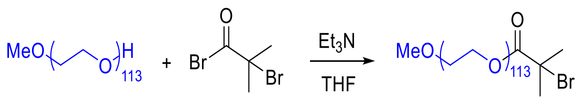

Scheme 1. Synthesis of mPEG-BiB.

mPEG-BiB was synthesised according to a literature procedure (Scheme 1).[4] Briefly, mPEG (5000 g mol^{-1}, 2.5 mmol) was dissolved in dry THF (100 mL) under an argon atmosphere and the solution was cooled on ice. Then, triethylamine (0.37 mL, 2.7 mmol, 1.1 eq) was added, followed by 2-bromoisobutyrylbromide (0.33 mL, 2.7 mmol, 1.1 eq). After overnight stirring, the product was filtered. The filtrate was concentrated at reduced pressure and the residue was redissolved in dichloromethane. The product was then isolated by precipitation with diethylether and then dried under vacuum.

BioPISA

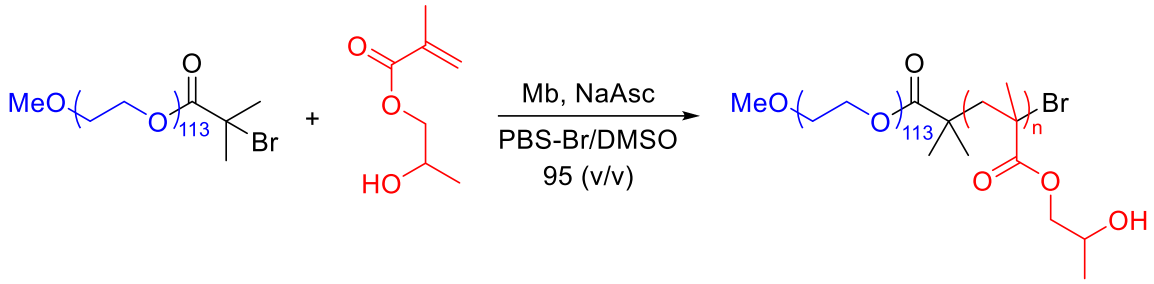

Scheme 2. Typical bioPISA reaction.

COMPLETE TECHNIQUES

NMR spectroscopy

NMR spectra were recorded on a Bruker Avance III 300 MHz NMR spectrometer (^{1}H NMR 300 MHz) or a Bruker Avance III 400 MHz NMR spectrometer (^{1}H NMR 400 MHz) using deuterated dimethyl sulfoxide (DMSO-d_6) as the solvent. Chemical shifts of protons are reported as δ in parts per million (ppm) and are relative to DMSO-d_6 at δ = 2.54 ppm. Data were evaluated with the MestReNova software suite (v 12.0).

Size exclusion chromatography

To analyse the resulting block copolymers by size exclusion chromatography (SEC), the DP 350, 20 wt% reaction mixtures was centrifuged, and the supernatant removed. The precipitate was diluted in THF. Copolymer samples were prepared as follow: the bioPISA suspension was centrifuged (11000 x g) and the supernatant was removed. The sample was resuspended in 18 mL DI water, and the purification was repeated 4 times. The remaining residue was let to dry overnight at 40 °C and then suspended in 2 mL THF. The supernatant was passed through a 0.2 µm syringe filter (Whatman® Puradisc 13, PP) before analysis by THF SEC.

SEC experiments were performed on an Agilent 1200 series HPLC system equipped with an Agilent PLgel mixed guard column (particle size= 5 µm) and two Agilent PLgel mixed-D columns (ID = 7.5 mm, L = 300 mm, particle size = 5 µm). Signals were recorded by a UV detector (Agilent 1200 series), an Optilab REX interferometric refractometer, and a miniDawn TREOS light scattering detector (Wyatt Technology Corp.). Samples were run using THF as the eluent at 30 °C and a flow rate of 1.0 mL min^{-1}. Data analyses were carried out on Astra software (Wyatt Technology Corp.) and molecular weights were determined based on narrow molecular weight polystyrene standards calibration (from 540 to 2'210'000 g mol^{-1}).

Dynamic light scattering (DLS)

DLS data were obtained with 2 ml of a dilute aqueous suspension. Data were collected at constant temperature (25 °C) on a commercial goniometer instrument (3D LS Spectrometer, LS Instruments AG, Switzerland) at angle 90°. The primary beam was formed by a linearly polarized and collimated laser beam (Cobolt 05-01 diode pumped solid state laser, λ = 660 nm, P_{max} = 500 mW), and the scattered light was collected by single-mode optical fibers equipped with integrated collimation optics. The incoming laser beam passed through a Glan-Thompson polarizer with an extinction ratio of 10^{-6}. Another Glan-Thompson polarizer, with an extinction ratio of 10^{-8}, was mounted in front of the collection optics. To construct the intensity auto-correlation function $g_2(t)$, the collected light was coupled into two APD detectors via laser-line filters (Perkin Elmer, Single Photon Counting Module), and their outputs were fed into a two-channel multiple-tau correlator. To improve the signal-to-noise ratio and to eliminate the impact of detector after-pulsing on $g_2(t)$ at early lag times below 1

µs, these two channels were cross-correlated. The field auto-correlation function was obtained *via* the Siegert relation: $g_1(\mathrm{t}) = \sqrt{g_2(t) - 1}$. The hydrodynamic radius (R_h) was determined from the Stokes-Einstein relation:

$$R_\mathrm{h} = \frac{k_\mathrm{B}T}{6\pi\eta D}$$

Where k_B is the Boltzmann constant, T the temperature, η the viscosity of the solvent, and *D* the diffusion coefficient was determined using a second order cumulant fit of $g_2(t)$. Because of the large size of the polymersomes, only the smaller spherical morphologies could be properly analysed via DLS.

For the characterization of Alexa 405 SiO_2NP, one drop of the suspension was diluted in deionised water (2 mL). DLS measurements of PISA assemblies were performed on a Zetasizer Nano ZSP (Malvern Instruments, UK) at 25 °C, 173°. Stock solutions were diluted 1:1000 in PBS in a cuvette, with a final volume of 1 mL, measured during 11 runs, repeated three times. Alexa 405-tagged SIO_2 NPs were instead measured according to the same protocol, on a Zetasizer Nano ZS90, at 25°C and 90°.

REACTION KINETICS

BioPISA reactions were prepared as described and DMSO was replaced by DMF to allow monitoring of the monomer concentration by NMR spectroscopy. Reaction mixtures samples (0.1 mL) were taken at t = 0 and every 10 min and diluted in DMSO-*d₆* (0.5 mL) before analysis by ^{1}H NMR spectroscopy.

centrifugation (500 x g, 3 min, in an Eppendorf microcentrifuge, 2x) and washing with PBS, and the resulting washing supernatant. Volumes of both GUVs and supernatant were kept constant.

CO-ENCAPSULATION OF GLUCOSE OXIDASE (GOX)

To obtain GOX-loaded vesicles, GOX was dissolved (1 mg mL^{-1}) together with Mb in the reaction mixture. Then, the vesicles were purified. If needed, samples were incubated 10 minutes in a 1 mg mL^{-1} NaAsc solution before being centrifuged and resuspended (2x) in PBS again.

Enzyme stability assay

In two separate vials, 10 mg of Mb or β-gal were dissolved in 2 mL PBS-Br. In one of the vials, 20 wt% HPMA and 5 v% DMSO substituted equivalent volumes of PBS-Br. The two vials were incubated at 37 °C under Ar for 2 h. Then, the enzymes mixed with monomer and DMSO were filtered with a 10 kDa spin diafiltration device (Amicon, Merck) to remove the small molecules, and resuspended in PBS-Br. The enzymatic activity assays were performed with equal volumes from the two vials; for Mb as described above, for β-gal FDG was substituted with the same quantity of *o*-nitrophenyl-β-galactoside and absorbance was measured at 405 nm for 10 minutes. The initial slope values of the kinetics were compared.

Enzyme labelling

10 mg of Mb and β-gal were each dissolved in 1 mL PBS pH 8.4, and mixed with 10 µl of 1 mM Atto 488-NHS ester in DMSO or 10 µl 10 MCy5-NHS ester in DMSO, respectively. After overnight reaction at 4°C, the enzymes were purified with a 10 kDa spin diafiltration device, and resuspended in PBS-Br. GUVs encapsulating both enzymes were prepared, with 10 wt% labelled enzyme.

ENZYME ACTIVITY OF ALKALINE PHOSPHATASE (ALP)

To obtain ALP-loaded vesicles, 10 µL of alkaline phosphatase (13 mg ml^{-1}) were added to the reaction mixture. Once purified, the enzymatic activity of β-gal was measured in a Clariostar Plus plate reader. 10 µL of vesicles were mixed with 2 µL *para*-nitrophenylphosphate (pNPP) 1 mM, to a final volume of 200 µL in Tris-HCl. The formation of para-nitrophenol was followed at absorbance 405 nm.

The first fraction was subjected to several centrifugation and washing steps. All resulting supernatants and GUVs were incubated with trypsin (final concentration 0.5 mg mL^{-1} into 100 µL of sample) for 2 hours at 37 °C, and the

resulting activities were compared to non-trypsinized samples. Volumes of both GUVs and supernatant were kept constant. The values were analysed with a t-test.

POLYMERIZATION IN PRESENCE OF BACTERIA

100 µL of *E. coli* BL21 (DE3) expressing mClover at OD_{600} 1 were added to the reaction mixture. After the polymerization, the monomer conversion yield was measured via NMR spectroscopy. To image the structures, the polymer membranes were stained with Cy5-$PEG_{3.5k}$-cholesterol. For viability measurements post polymerization, non-fluorescent *E. coli* were incubated with a final 0.5 mM of fluorescein diacetate; 1 µM propidium Iodide (PI) was selected as a stain for dead cells, and substituted Cy5-$PEG_{3.5k}$-cholesterol. 100 cells per conditions were imaged (either green or red fluorescent). Bacteria incubated at 95 °C for 10 minutes were the negative control.

FLOW CYTOMETRY

GUVs encapsulating the cell free expression kit and either the plasmid pNCS-mClover3, Chx10 3kb AP (alkaline phosphatase vector) or no plasmid at all were produced. To 35 µL of sample, 100 µl of S30 premix and 30 µL of complete amino acid mixture were added. The samples were incubated at 37 °C for 4 h.

For cytometric analysis of the vesicles, the CytoFLEX S by Beckman Coulter was used (CytExpert Version 2.4.0.28). Samples were diluted prior to measurement with PBS to achieve an abort rate below 10%. To account for small particle size compared to cells, the primary threshold (trigger level) and the width channel were set to SSC (height: 1000). 50000 events per sample were recorded and a gating strategy was applied to exclude debris (SSC-A [log] vs FSC-A [log]) and to analyse only single vesicles per event (SSC-A [log] vs SSC-Width [linear]). Fluorescence of mClover was measured in the FITC channel (525/40 nm) at maximum gain due to small particle size.

TRANSMISSION ELECTRONIC MICROSCOPY

Dry-state-stained transmission electron microscopy (TEM) imaging was performed on Tecnai Spirit transmission electron microscope (TEM, FEI/ThermoFisher, Hillsboro, Oregon, United States) operating at 120 kV using a 2k Veleta camera (Olympus, Shinjuku, Tokyo, Japan). All dry-state samples were diluted with deionized water to appropriate analysis concentration and then deposited onto GO-coated copper grids. After roughly 1 min, excess sample was blotted from the grid and the grid was stained with methylamine vanadate (Nanovan) negative stain solution for 1 min prior to blotting, drying and microscopic analysis.

CRYO-TRANSMISSION ELECTRONIC MICROSCOPY

A 4 µl aliquot of sample was adsorbed onto holey carbon-coated grid (Lacey, Tedpella, USA), blotted with Whatman 1 filter paper and vitrified into liquid ethane at -180 °C using a Leica GP2 plunger (Leica microsystems, Austria). Frozen grids were transferred onto a Talos Electron microscope (FEI, USA) using a Gatan 626 cryo-holder (GATAN, USA). Electron micrographs were recorded at an accelerating voltage of 200 kV using a low-dose system (40 e- $Å^{-2}$) and keeping the sample at -175 °C. Defocus values were -2 to 3 µm. Micrographs were recorded on 4K x 4K Ceta CMOS camera.

MORPHOLOGICAL CHARACTERIZATION OF GUVS

The cryoTEM micrographs of vesicles DP 300, 20 wt% were observed to determine the fraction presenting internal structures.

For the other characterizations, CLSM micrographs of GUVs were characterized using the particle analysis tool in Image. The area was the area of the GUVs in their specific picture, as close as possible to their mid-section area. The aspect ratio was defined as the ratio between minimum and maximum Feret diameter (i.e., the calliper diameters), where a non-perfectly isometric figure has aspect ratio <1. The vesicle fluorescence intensity was

divided by its area, to control for higher cumulative intensities resulting from larger vesicles. The filling ratio was the name given to the solidity parameter, defined as the ratio of the area of the vesicle to the area of its convex hull. The convex hull is the smallest convex shape that can enclose the vesicle. A perfectly homogeneous round object has solidity = 1, whereas objects with concavities or lower compactness (e.g., holes) will have lower values. The fluorescence signal of Actin-Atto 488 or Phalloidin-Atto565 were used to determine solidity.

DATA ANALYSIS

Numerical data deriving from kinetic runs and vesicle characterizations were analysed and plotted with OriginLab 2021 and Graphpad Prism 9.

SUPPORTING INFORMATION

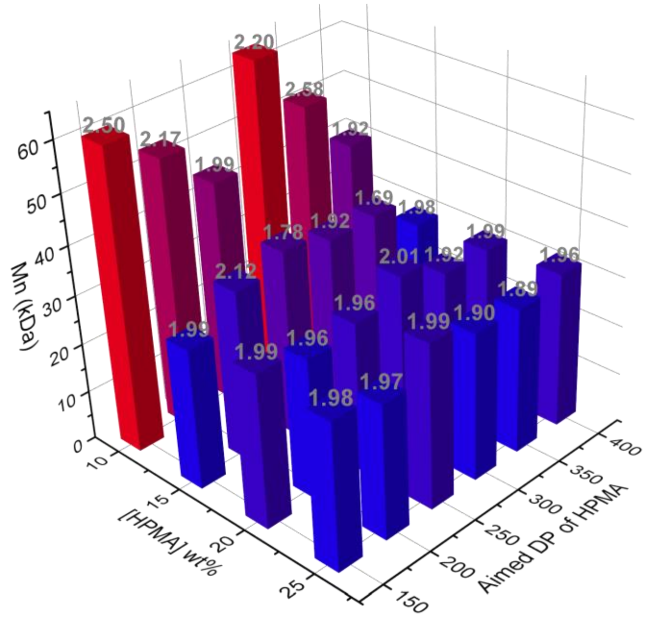

Figure S1. Molecular weight and *Đ* determined by SEC (THF, 30 °C, PS standard) of the soluble fraction of the resulting copolymers depending on the initial [HPMA] and aimed DP for bioPISA in PBS-Br. Note: as substantial parts of the polymer were not soluble in common solvents, and because the the molecular weight of the soluble polymer fractions were around 30 kDa for [HPMA] > 10wt%, this suggests that the aimed DP is not the real DP and the important parameter could be the initiator concentration as was demonstrated for RAFT by Khor *et al*.[5]

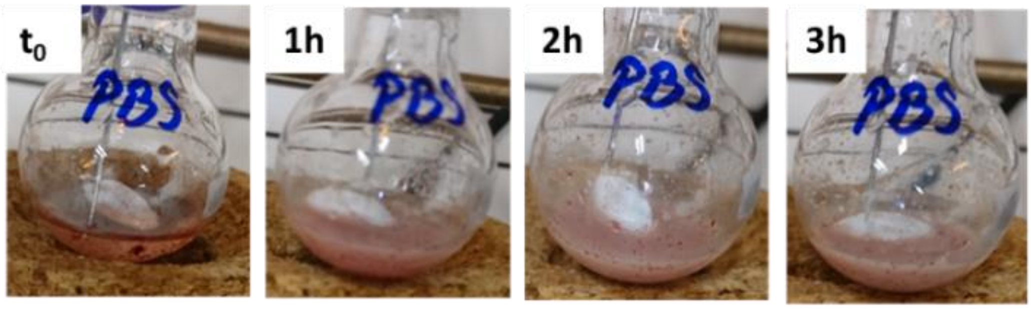

Figure S2. Photos of a bioPISA reaction mixture (DP 150 10 wt%) taken over time. The progressive increase in turbidity is due to the formation of nanostructures.

Reaction conditions

In control reactions, no reaction occurred without monomer, whereas reactions in which either mPEG-BiB, NaAsc, or Mb were omitted proceeded with a low monomer consumption of <20%. Thus, a low level of blank reaction happened, but the initiator, oxidising agent, and the metallo-protein are necessary for the reaction to proceed to high conversions (**Table S1**). The organic co-solvent DMSO was necessary as well, likely as solubilizer for the growing chains.[6,7] Furthermore, polymerisations were unsuccessful when they were carried out in pure PBS or in a 100 mM NaBr solution in deionised water, indicating that both the salts in the PBS buffer and the NaBr were required for the polymerisation to happen, most likely because the buffer maintained the pH and NaBr maintained an excess of halides that is need for efficient ATRP in aqueous conditions.[8]

Table S1. Details of the bioPISA reactions conditions

**Experiments**		**Conditions**					
**[HPMA] (wt%)**	**Aimed HPMA (DP)**	**mPEG-Bib (mg)**	**Mb (mg)**	**HPMA (µL)**	**NaAsc (mg)**	**DMF (mL)**	**PBS (mL)**
10	150	46 (1 eq)	4.7 (0.03 eq)	187 (150 eq)	11.9 (6 eq)	0.10	1.70
10	200	35 (1 eq)	3.6 (0.03 eq)	187 (200 eq)	8.3 (6 eq)	0.10	1.70
10	250	30 (1 eq)	3.1 (0.03 eq)	187 (250 eq)	7.4 (6 eq)	0.10	1.70
10	300	25 (1 eq)	2.6 (0.03 eq)	187 (300 eq)	5.9 (6 eq)	0.10	1.70
10	350	20 (1 eq)	2.0 (0.03 eq)	187 (350 eq)	4.8 (6 eq)	0.10	1.70
10	400	17 (1 eq)	1.7 (0.03 eq)	187 (400 eq)	4.0 (6 eq)	0.10	1.70
15	150	73 (1 eq)	7.4 (0.03 eq)	296 (150 eq)	17.6 (6 eq)	0.10	1.70
15	200	55 (1 eq)	5.6 (0.03 eq)	296 (200 eq)	13.1 (6 eq)	0.10	1.70
15	250	44 (1 eq)	4.5 (0.03 eq)	296 (250 eq)	10.5 (6 eq)	0.10	1.70
15	300	29 (1 eq)	3.8 (0.03 eq)	296 (300 eq)	12.0 (6 eq)	0.10	1.70
15	350	31 (1 eq)	3.2 (0.03 eq)	296 (350 eq)	7.4 (6 eq)	0.10	1.70
15	400	27 (1 eq)	2.8 (0.03 eq)	296 (400 eq)	6.4 (6 eq)	0.10	1.70
20	150	102 (1 eq)	10.4 (0.03 eq)	414 (150 eq)	24.3 (6 eq)	0.10	1.70
20	200	77 (1 eq)	7.9 (0.03 eq)	414 (200 eq)	18.3 (6 eq)	0.10	1.70
20	250	61 (1 eq)	6.2 (0.03 eq)	414 (250 eq)	14.5 (6 eq)	0.10	1.70
20	300	51 (1 eq)	5.1 (0.03 eq)	414 (300 eq)	12.1 (6 eq)	0.10	1.70
20	350	44 (1 eq)	4.5 (0.03 eq)	414 (350 eq)	10.5 (6 eq)	0.10	1.70
20	400	39 (1 eq)	4.0 (0.03 eq)	414 (400 eq)	9.3 (6 eq)	0.10	1.70
25	150	136 (1 eq)	13.9 (0.03 eq)	552 (150 eq)	32.3 (6 eq)	0.10	1.70
25	200	102 (1 eq)	10.4 (0.03 eq)	552 (200 eq)	24.3 (6 eq)	0.10	1.70
25	250	82 (1 eq)	8.4 (0.03 eq)	552 (250 eq)	19.5 (6 eq)	0.10	1.70
25	300	68 (1 eq)	6.5 (0.03 eq)	552 (300 eq)	16.2 (6 eq)	0.10	1.70
25	350	58 (1 eq)	5.9 (0.03 eq)	552 (350 eq)	13.8 (6 eq)	0.10	1.70
25	400	51 (1 eq)	5.2 (0.03 eq)	552 (400 eq)	12.1 (6 eq)	0.10	1.70

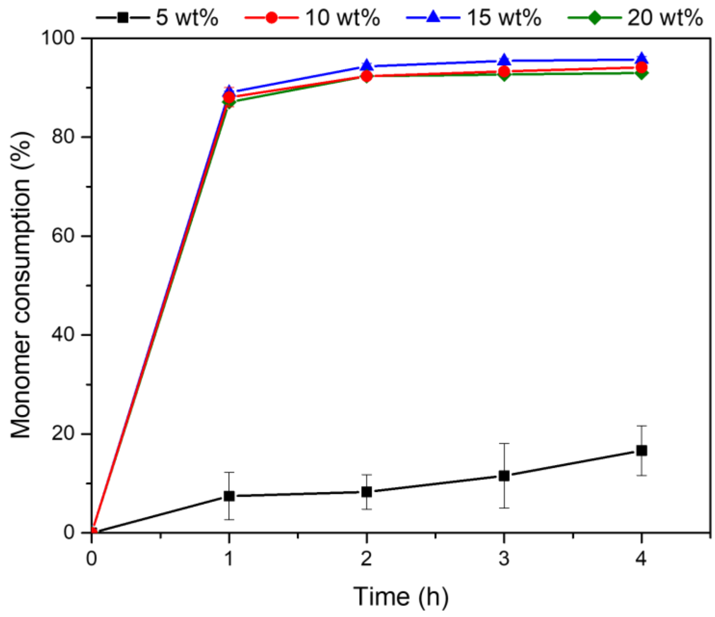

Figure S3. Monomer consumption over time ([HPMA] = 20 wt% and aimed DP = 150) at different initial monomer concentrations: 5 (black squares), 10 (red circles), 15 (blue triangles) and 20 (green diamonds) wt% with a HPMA aimed DP of 150. Error bars as mean ± SD, n = 3 polymerizations.

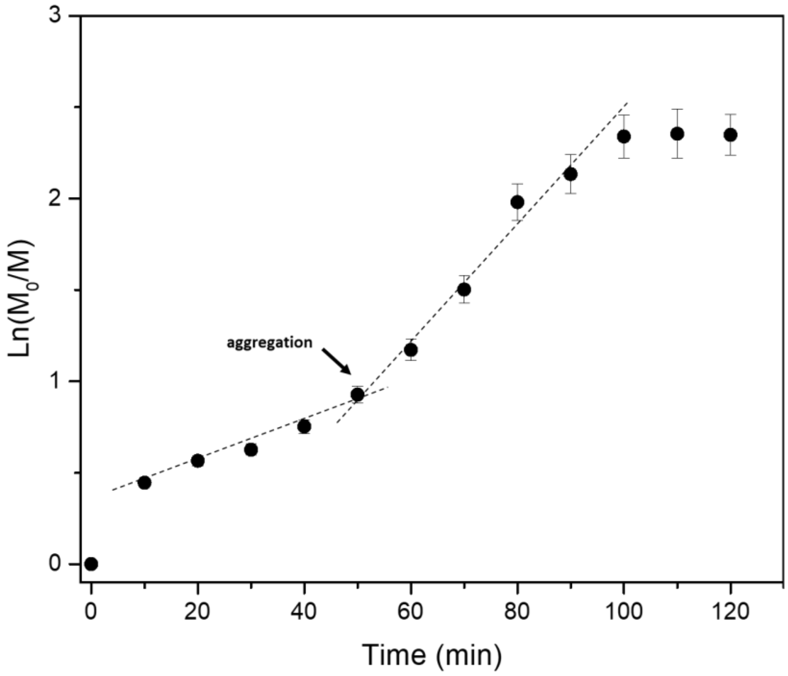

Figure S4. Kinetic data of bioPISA (DP 300 20 wt%) with 10 min intervals showing the onset of aggregation after 50 min of reaction before reaching a plateau at 100 min . Error bars as mean ± SD, n = 3 polymerizations.

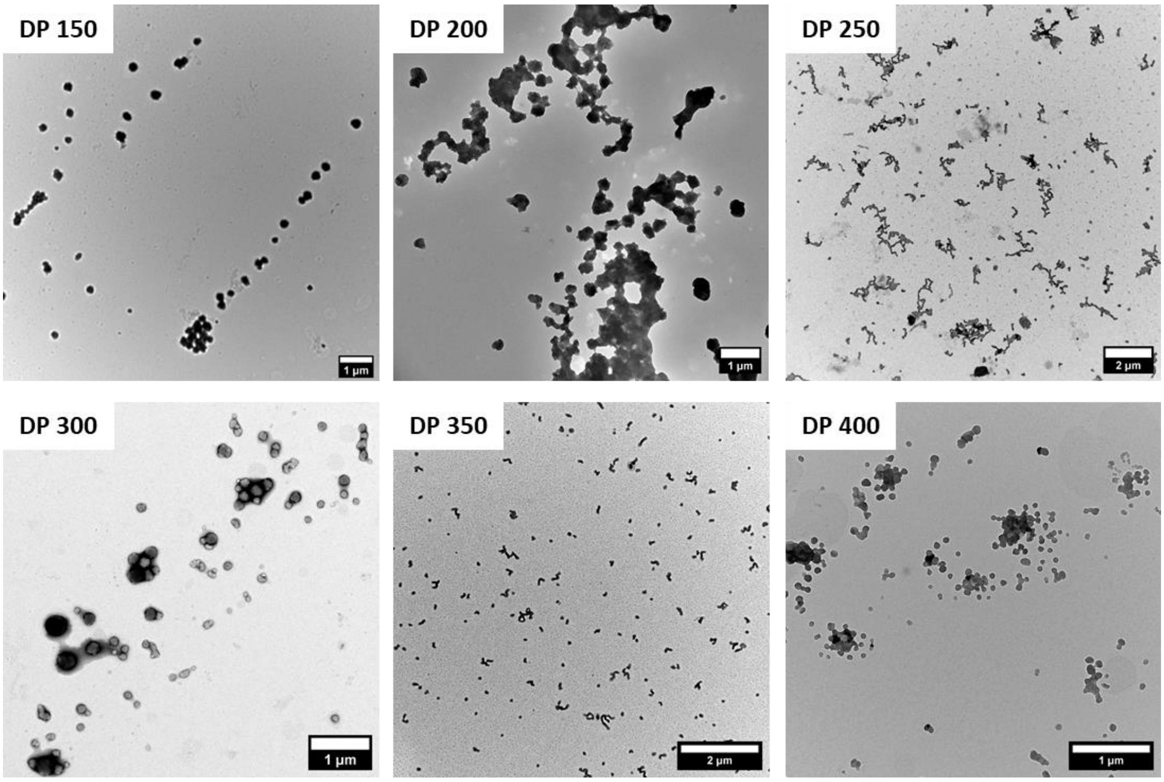

Figure S5. TEM images of morphologies from bioPISA at [HPMA] = 10 wt% and aimed DP = 150, 200, 250, 300, 350 and 400. Scalebars: 1 µm (DP = 150, 200, 300, 400), 2 µm (DP = 250, 350).

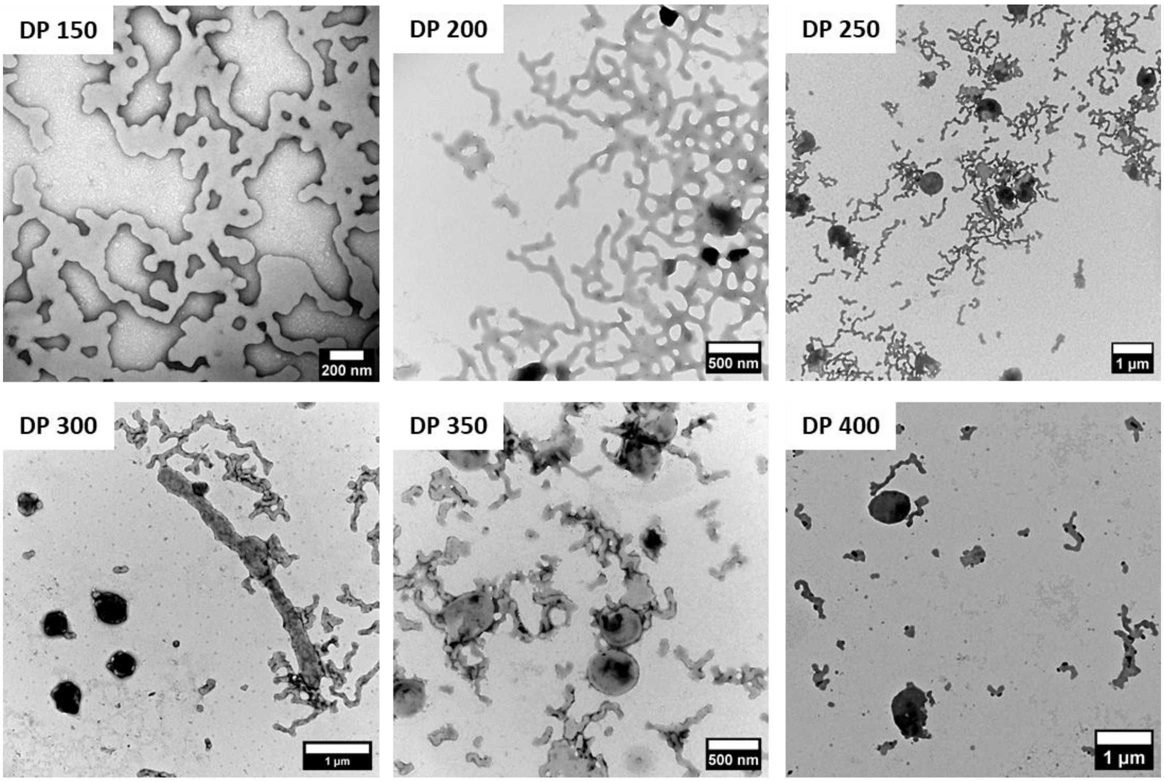

Figure S6. TEM images of morphologies from bioPISA at [HPMA] = 15 wt% and aimed DP = 150, 200, 250, 300, 350 and 400. Scalebars: 200 nm (DP = 150), 500 nm (DP = 200, 350), 1 µm (DP = 250, 300, 400).

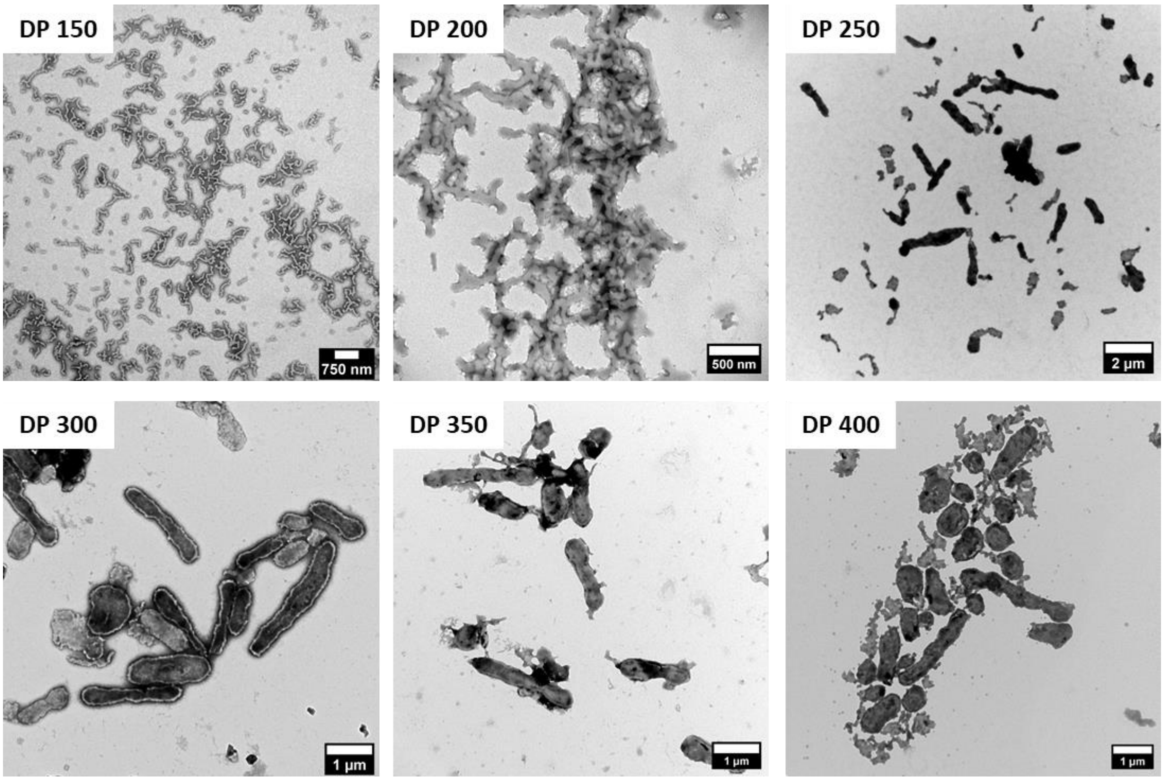

Figure S7. TEM images of morphologies from bioPISA at [HPMA] = 20 wt% and aimed DP = 150, 200, 250, 300, 350 and 400. Some drying artifacts (dots, patches) can be seen. Scalebars: 500 nm (DP = 200), 750 nm (DP = 150), 1 µm (DP = 300, 350, 400). 2 µm (DP = 250).

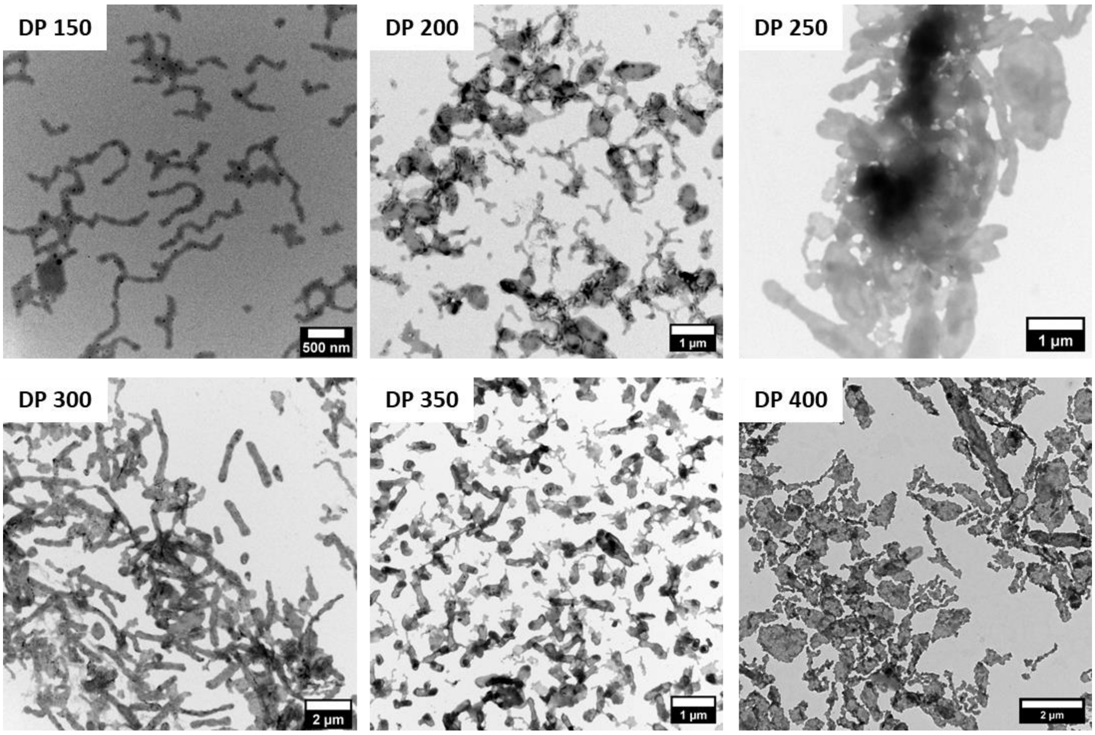

Figure S8. TEM images of morphologies from bioPISA at [HPMA] = 25 wt% and aimed DP = 150, 200, 250, 300, 350 and 400. Some drying artifacts (dots, patches) can be seen. Scalebars: 500 nm (DP = 150), 1 µm (DP = 200, 250, 350), 2 µm (DP = 300, 400).

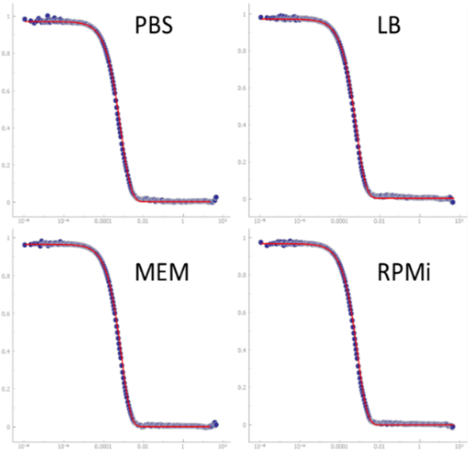

Solvent	D_h (nm)	*PDI*
PBS	238 ±22	0.28
LB	242 ±30	0.15
MEM	234 ±23	0.12
RPMi	208 ±24	0.12

[a] DLS 90 ° @ 25 °C, 10 × 60 s measurements. Cumulant 2nd.

Figure S9. DLS analysis (correlograms and numerical values) of the spherical nanostructures synthesised by bioPISA in PBS-Br at [HPMA] 10 wt% and aimed DP 150.

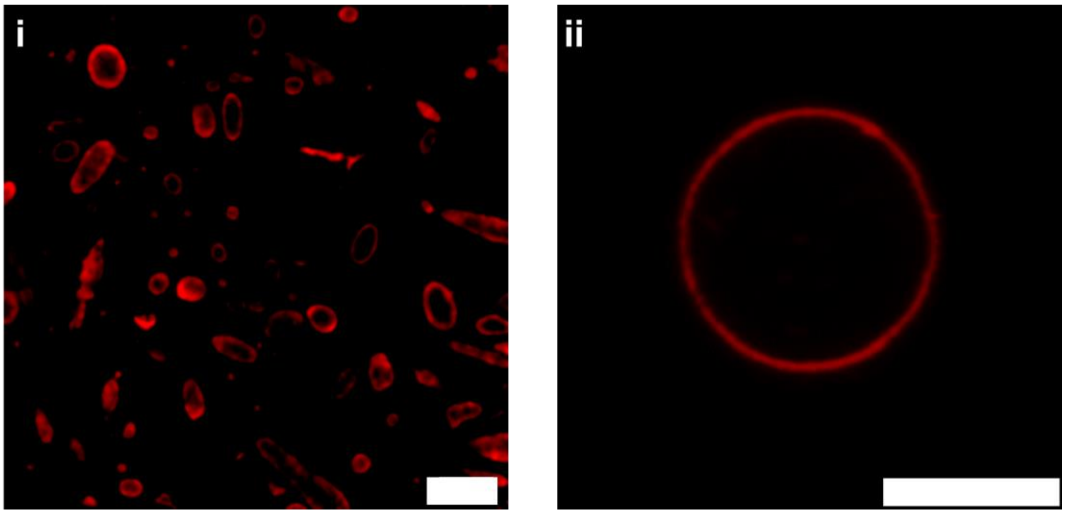

Figure S10. CLSM micrographs of GUVs obtained from bioPISA, aimed DP 400, 20 wt% (red: Cy5-PEG$_{3.5k}$-cholesterol). The different thicknesses of red staining are to be ascribed to internal structures. Scalebars: i: 10 µm, ii: 5 µm.

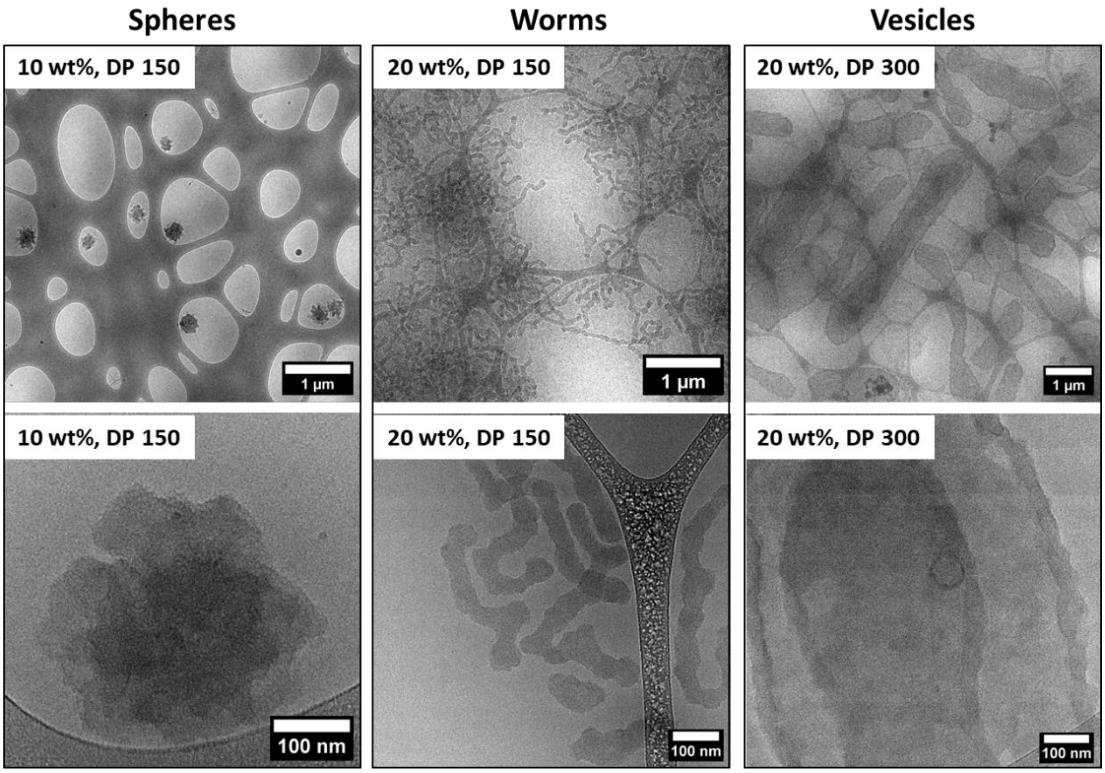

Figure S11. Cryo-TEM images of the three morphologies obtained by bioPISA in PBS-Br at [HPMA] = 10 wt% and aimed DP = 150 for the spheres (full), [HPMA] = 20 wt% and aimed DP = 150 for the worms and [HPMA] = 20 wt% and aimed DP = 300 for the vesicles (internal cavity). Scalebars, top row: 1 µm; bottom row: 100 nm.

SIZE EXCLUSION CHROMATOGRAPHY

The polymer was only partially soluble in the organic solvent. Several other solvents (DMF, DMSO, CH_2Cl_2, $CHCl_3$) were employed, but none achieved complete dissolution of the copolymer precipitate. It is hypothesised that cross-linking happened during the chain-extension, possibly due to transesterification of the side chains once inaccessible to the catalyst, which rendered part of the final copolymer insoluble.[9,10] The fraction of insoluble copolymer was 62% ± 8% irrespective of the molecular weight of the formed polymers or the morphology of the self-assemblies. The soluble parts of the bioPISA-derived diblock copolymers had number average molecular weights (M_n) of 30-60 kDa and dispersities ($Đ$) of 1.5-2.5 (**Figure S12** and **Table S2**). One explanation for the broad molecular weight distributions is that, unlike bioRAFT where the polymer chain does not need proximity to the enzyme to grow, ATRP necessitates the catalyst for the atom transfer (in this case Mb) to be close to the active polymer chain-end.[11] In PISA, the growing chain-ends are located in the hydrophobic core or membrane of the self-assemblies, and therefore difficult for the water-soluble enzyme to reach. This most likely led to a reduction in the degree of control of the polymerization and, as a result, in high dispersities. Thus, most likely the reaction proceeded through an ATRP-like initiation and initial RDRP until the block copolymers become water-insoluble, followed by a free radical polymerisation inside of the hydrophobic parts of the formed self-assemblies. While this might seem to be a disadvantage, it did not significantly affect the predictability of our method in generating a variety of self-assembled nanostructures, as discussed above.

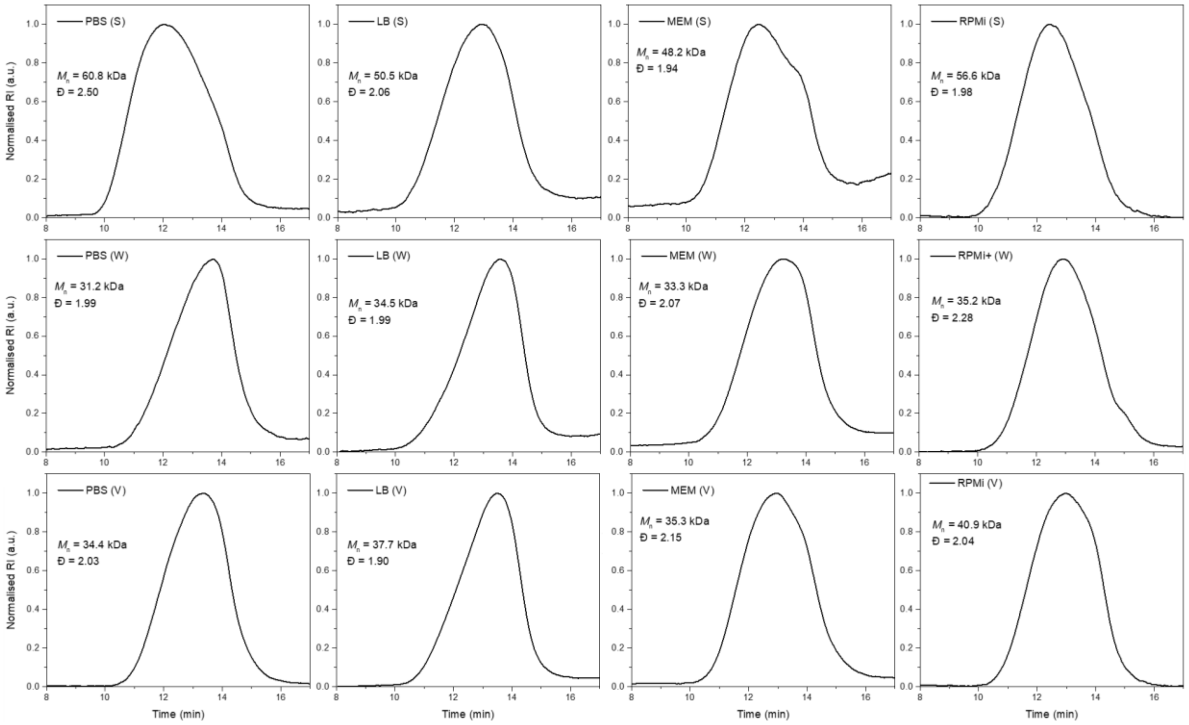

Figure S12. SEC (THF, 30 °C, PS standard) chromatogram of the soluble fraction of mPEG-b-PHPMA copolymers from different morphologies (spheres: [HPMA] = 10 wt% and aimed DP = 150, worms: [HPMA] = 20 wt% and aimed DP = 150, vesicles: [HPMA] = 20 wt% and aimed DP = 300) synthesised by bioPISA in different media (PBS, LB, MEM and RPMi).

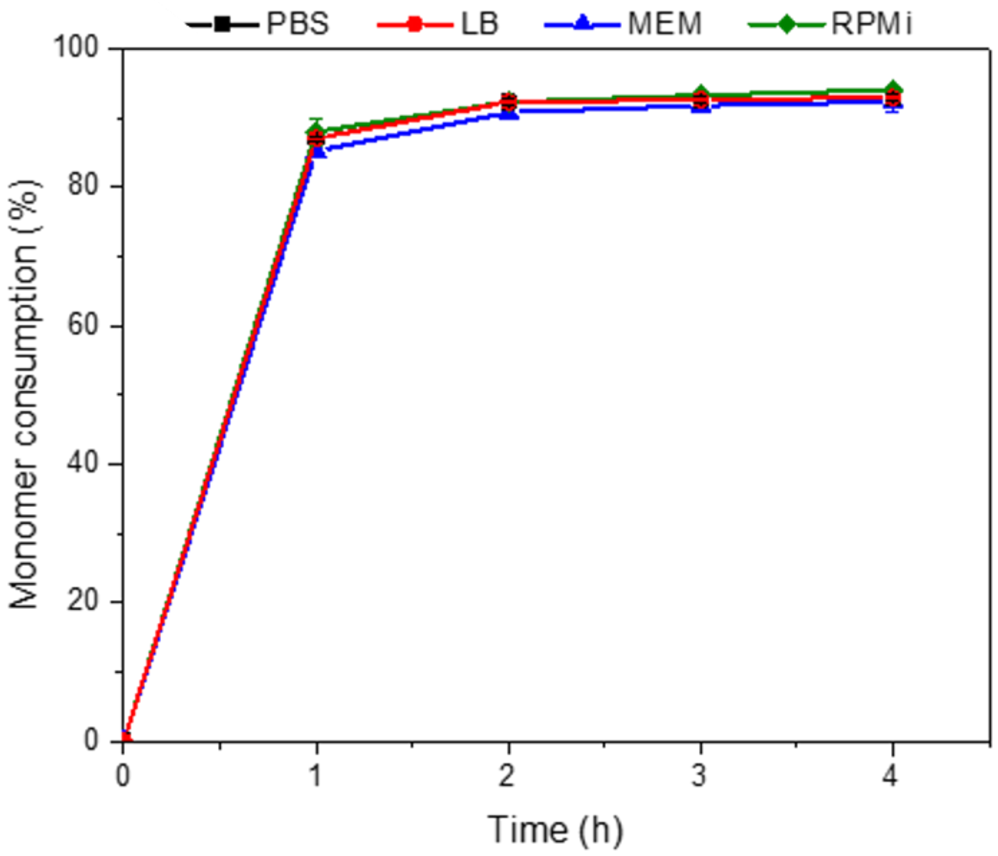

Figure S13. Monomer consumption over time ([HPMA] = 20 wt% and aimed DP = 150) in different media: PBS (black squares), LB broth (red circles), MEM (blue triangles) and RPMi-1640 (green diamonds). Error bars as mean ± SD, n = 3 polymerizations.

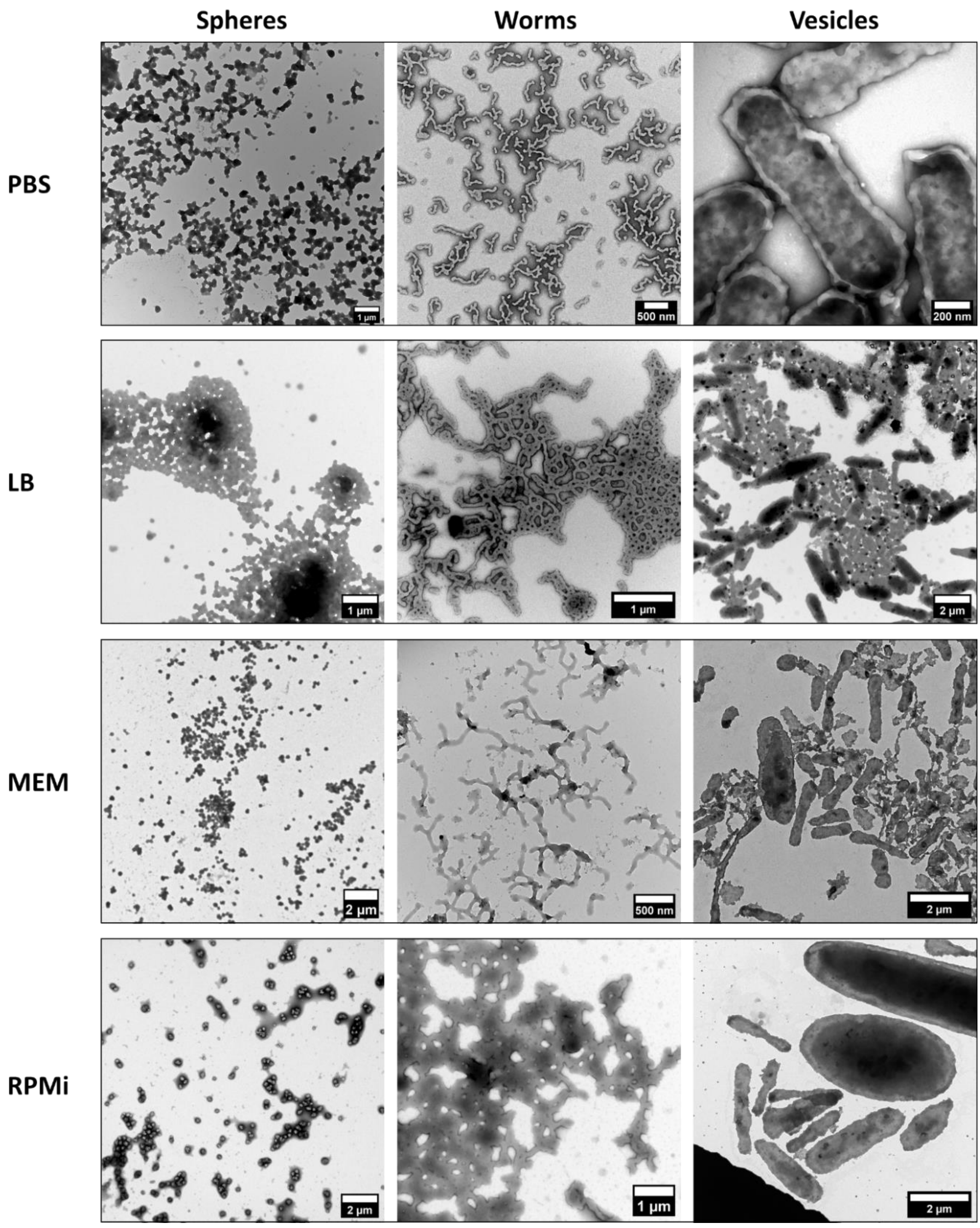

Figure S14. TEM images of the three morphologies obtained by bioPISA in different complex aqueous media (PBS, LB, MEM, RPMi) at [HPMA] = 10 wt% and aimed DP = 150 for the spheres, [HPMA] = 20 wt% and aimed DP = 150 for the worms and [HPMA] = 20 wt% and aimed DP = 300 for the vesicles. Scalebars, left to right, top to bottom: 1 µm, 500 nm, 200 nm, 1 µm, 1 µm, 2 µm, 2 µm, 500 nm, 2 µm, 2 µm, 1 µm, 2 µm, respectively.

BIOCOMPATIBILITY AND BACTERIAL ENCAPSULATION

To test the compatibility of bioPISA with living cells, bioPISA reactions were performed in the presence of bacteria. After bioPISA the bacteria were both inside and outside of the self-assembled polymer structures. Although either the interactions of *Escherichia coli* with the polymer chains, or the extracellular environment altered the self-assembly process, resulting in filled microparticles (Figure S15), we could observe spatial association of the bacteria with the polymer and encapsulation of the bacteria within the polymer structures. Importantly, the live/dead assay for bacteria showed a good viability (~70%) of these cells despite the use of an oxygen-free buffer that furthermore did not contain any nutrients and was rich in bromide ions, suggesting the biocompatibility of bioPISA (Figure S16).

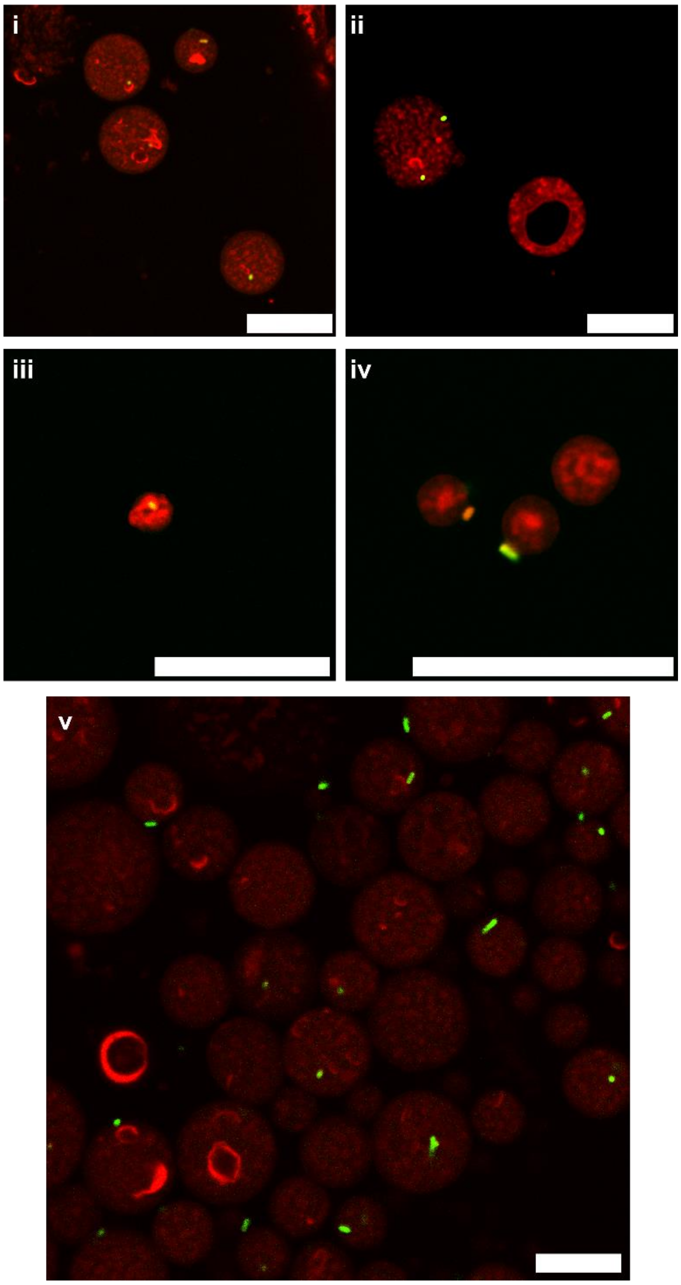

Figure S15. i-v) Several CLSM micrographs of bioPISA-derived microparticles containing fluorescent *E. coli*. (Green: mClover expressed in *E. coli*, red: Cy5-PEG$_{3.5k}$-cholesterol). Scalebars for all images: 20 µm.

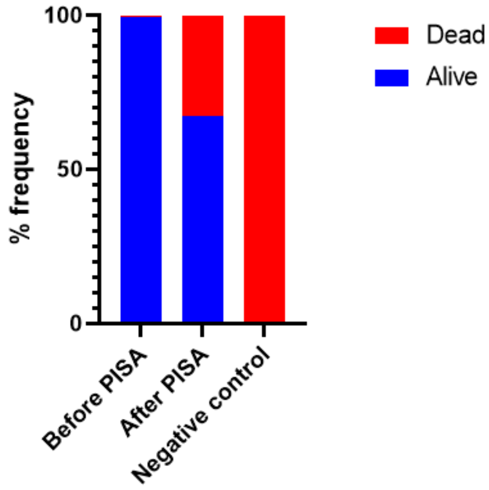

Figure S16. Live/dead assay of bacteria, showing their survival during bioPISA in PBS-Br. The negative control was provided by cells incubated at 95 °C for 10 minutes.

Reaction overview

Table S2. Overview of the bioPISA reactions and copolymers characterisation

[HPMA] (wt%)	*Aimed HPMA DP*	*Solvent*	M_n (kDa)[a]	*Đ*[a]	*Monomer consumption (%)*[b]	*Morphology*[c]
5	150	PBS	-	-	17	-
5	200	PBS	-	-	44	-
5	250	PBS	-	-	39	-
5	300	PBS	-	-	44	-
5	350	PBS	-	-	56	-
5	400	PBS	-	-	53	-
10	150	PBS	-	-	13[d]	-
10	150	PBS	-	-	15[e]	-
10	-	PBS	-	-	12[f]	-
10	150	PBS	60.8	2.50	95	Spheres
10	150	LB	50.5	2.06	94	Spheres
10	150	MEM	48.2	1.94	88	Spheres

10	150	RPMi	56.6	1.98	92	Spheres
10	200	PBS	54.2	2.17	92	Mixed
10	250	PBS	45.2	1.99	95	Mixed
10	300	PBS	64.6	2.20	78	Spheres
10	350	PBS	51.9	2.58	60	Mixed
10	400	PBS	40.0	1.92	38	Mixed
15	150	PBS	28.5	1.99	96	Worms
15	200	PBS	34.8	2.12	92	Worms
15	250	PBS	37.7	1.78	87	Mixed
15	300	PBS	35.3	1.92	94	Mixed
15	350	PBS	35.5	1.69	86	Mixed
15	400	PBS	29.1	1.98	39	Mixed
20	150	PBS	31.2	1.99	93	Worms
20	150	LB	35.0	1.98	93	Worms
20	150	MEM	33.3	2.07	93	Worms
20	150	RPMi	35.0	2.09	95	Worms
20	200	PBS	28.5	1.96	94	Worms
20	250	PBS	30.2	1.96	91	Mixed
20	300	PBS	34.4	2.01	95	Vesicles
20	300	LB	34.7	1.91	90	Vesicles
20	300	MEM	35.3	2.15	90	Vesicles
20	300	RPMi	37.6	2.05	95	Vesicles
20	350	PBS	30.0	1.92	96	Vesicles
20	400	PBS	30.0	1.99	95	Vesicles
25	150	PBS	29.9	1.98	93	Worms
25	200	PBS	27.3	1.97	94	Mixed
25	250	PBS	33.4	1.99	94	Vesicles
25	300	PBS	29.9	1.90	95	Vesicles
25	350	PBS	29.5	1.89	95	Vesicles
25	400	PBS	31.4	1.96	95	Vesicles

[a] Determined by SEC (THF, 30 °C, PS standard). [b] Determined by ^{1}H NMR spectroscopy (DMSO-d_6). [c] Determined by dry-state TEM microscopy. [d] Control reaction without NaAsc. [e] Control reaction without Mb. [f] Control reaction without mPEG-Bib.

ENCAPSULATION AND MEMBRANE PERMEABILITY

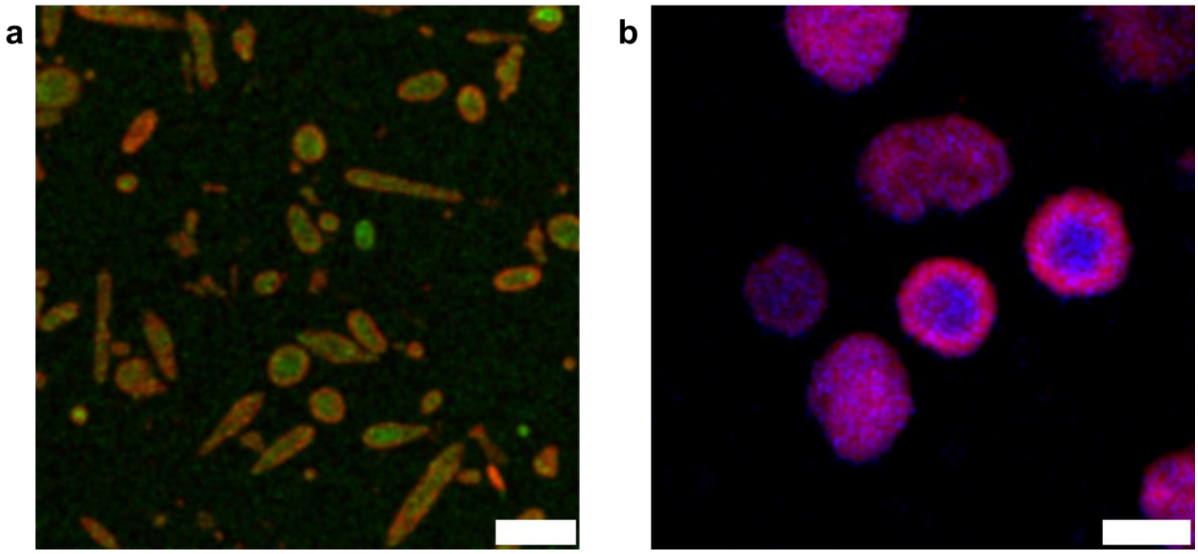

Figure S17. Overview CLSM micrographs of GUVs encapsulating cargo. a) FITC-Dextran 40 kDa, scalebar: 10 µm. b) Alexa 405 SiO2 NP, scalebar: 5 µm. Green: FITC, blue: Alexa 405; red: Cy5-PEG$_{3.5k}$-cholesterol.

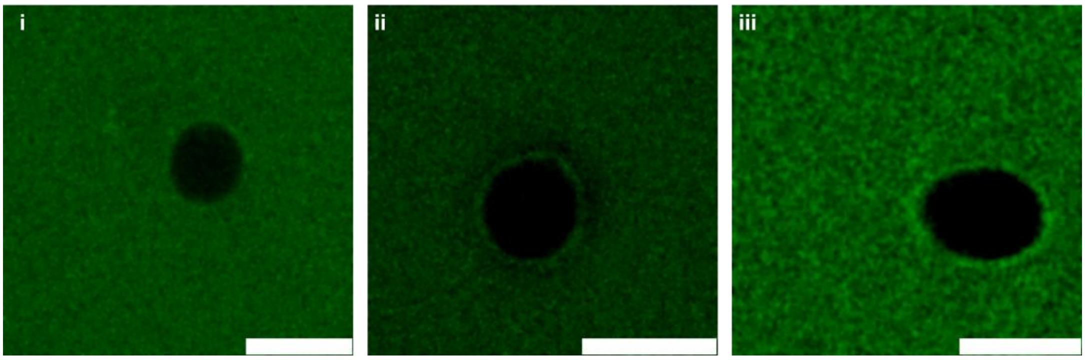

Figure S18. i-iii) Several CLSM micrographs of GUVs in a solution of FITC-Dextran 40 kDa. The vesicles are not fluorescent due to the impermeability of the membrane for the fluorescent dye (Green: FITC-Dextran 40 kDa). Scalebars for all images: 10 µm.

PURIFICATION OF GUVS VIA SIZE-EXCLUSION CHROMATOGRAPHY

Short PD 10 desalting columns have a claimed retention cut-off of 5000 Da. However, they were also previously reported to be able to separate low-concentration of proteins from vesicles.[12] To test whether they could be used for the separation of GUVs and cargo proteins, we recovered the first fraction (vesicles), washed it 1x with PBS, and then measured the Mb activity of the fractions before and after the washing steps, showing that the washed Mb-loaded GUVS had a similar activity than the original 1st fraction while the supernatant after washing had a much lower activity compared to the washed vesicles (**Figure S19**a). Thus, active protein of the 1st fraction resided in the vesicles and not in the surrounding solution. Similarly, the protein content in the vesicle-free supernatant was much lower than in the vesicles (**Figure S19**b). Finally, GUVs encapsulating ALP were washed during 4 cycles, and incubated with trypsin (0.5 mg mL^{-1}) for 1 hour. Both vesicles before and after washing retained their activity regardless of trypsinization, whereas the little activity detected in supernatants

was annihilated with enzymatic digestion (**Figure S19**c), thus confirming the successful purification of free enzyme from the GUVs.

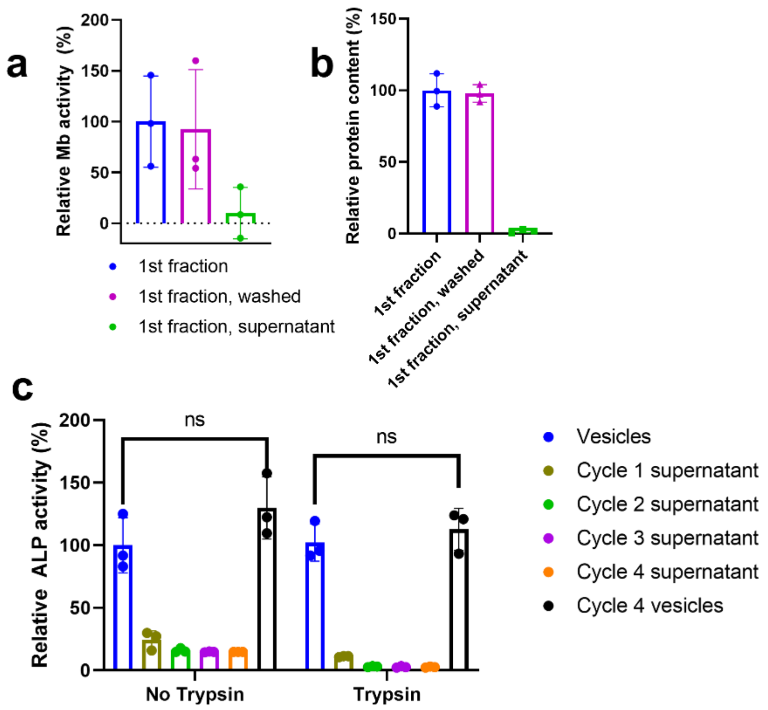

Figure S19. Purification of GUVs using PD 10 desalting columns. a) Relative Mb activity (oxidation of Ampex Red) from GUVs, GUVs after washing and resulting supernatant. b) Relative protein content from GUVs, GUVs after washing and resulting supernatant. c) Relative ALP activity (production of pNP from pNPP) of several fractions, with and without trypsin treatment. Mean ± SD, n=3 samples (n.s: p-value = 0.195, p-value = 0.486 respectively). Unpaired t-tests, multiple comparison with false discovery rate, two-stage step-up.

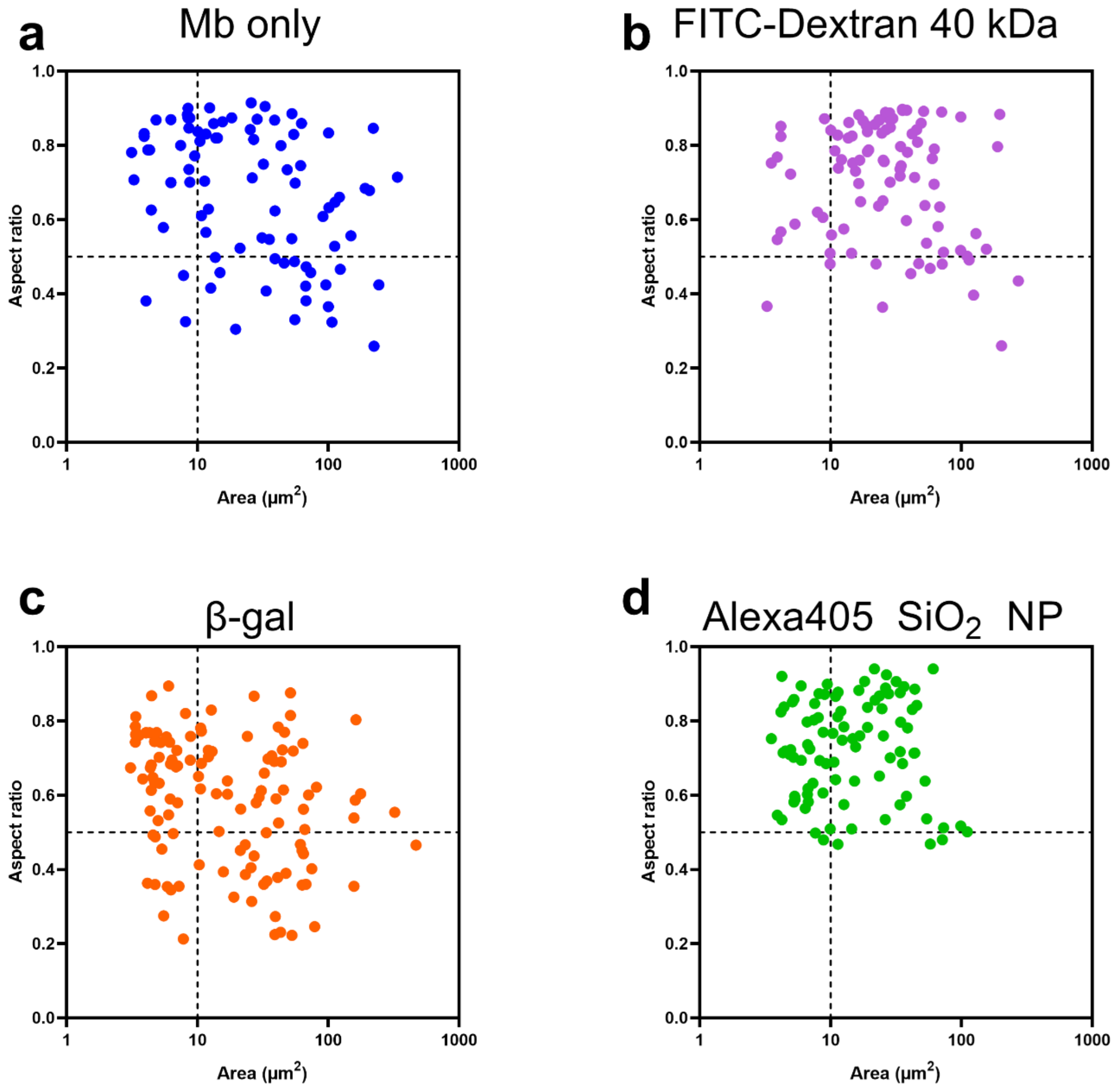

Figure S20. Scatter plots of the relation between size of GUVs and their aspect ratio. a) Mb-only loaded GUVs (r^2 = 0.07351, p-value = 0.0106, n = 88). b) GUVs with FITC-Dextran 40 kDa (r^2 = 0.09619, p-value = 0.0026, n = 92). c) GUVs loaded with the enzyme β-gal (r^2 = 0.02550, p-value = 0.0815, n = 120). d). GUVs loaded with Alexa405 SiO2 NP (r^2 = -0.1851, p-value = 0.0774, n = 92). The additional line at 10 μm^2 indicates an arbitrary delimitation between smaller and larger GUVs; the line at aspect ratio 0.5 indicates an arbitrary delimitation between rounder and more elongated shapes. r^2 calculated with Pearson correlation coefficients, two-tailed t-test.

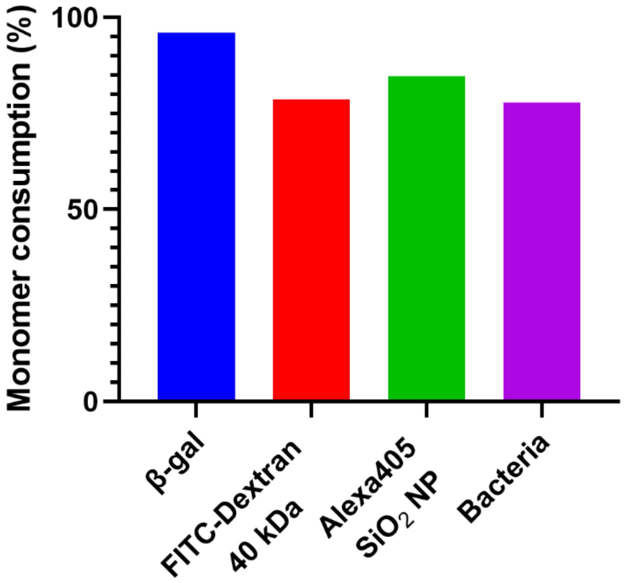

Figure S21. Monomer consumption during bioPISA in the presence of a selection of cargoes, showing the general tolerance of bioPISA for these bulky payloads.

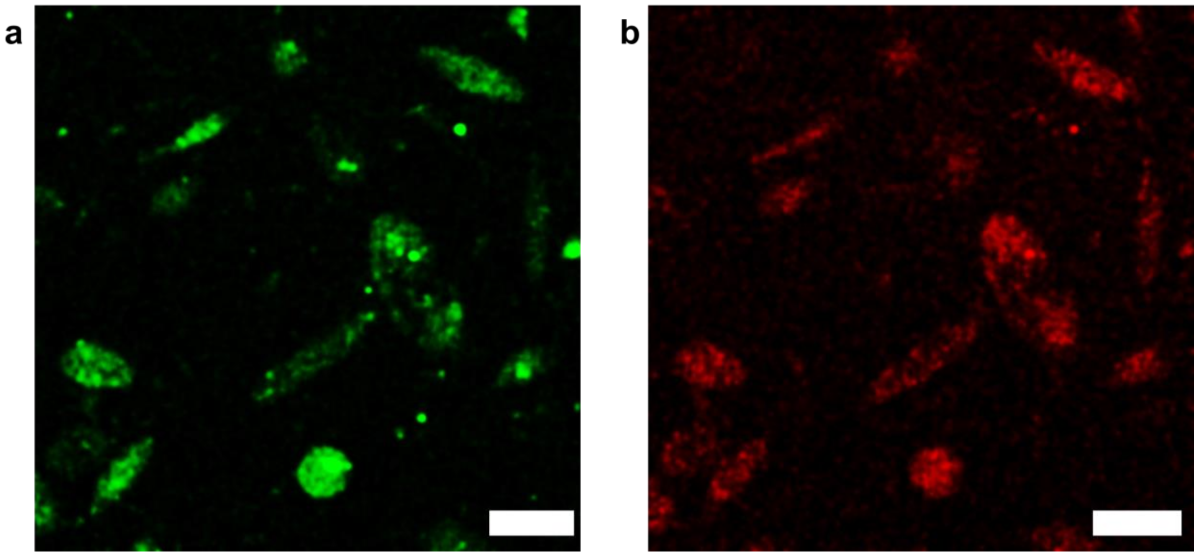

Figure S22. Overview CLSM micrographs **of** GUVs encapsulating both ATTO 488-labelled Mb (a) and Cy5-labelled β-gal (b). The fluorescence is mostly homogenously distributed througout the GUVs and, thereby, througout the lumen of the GUV, but some brighter spots (possibly aggregates) can be observed within the vesicles. Please note that the polymersome membrane was not stained in these images. Scalebars for both images: 10 µm.

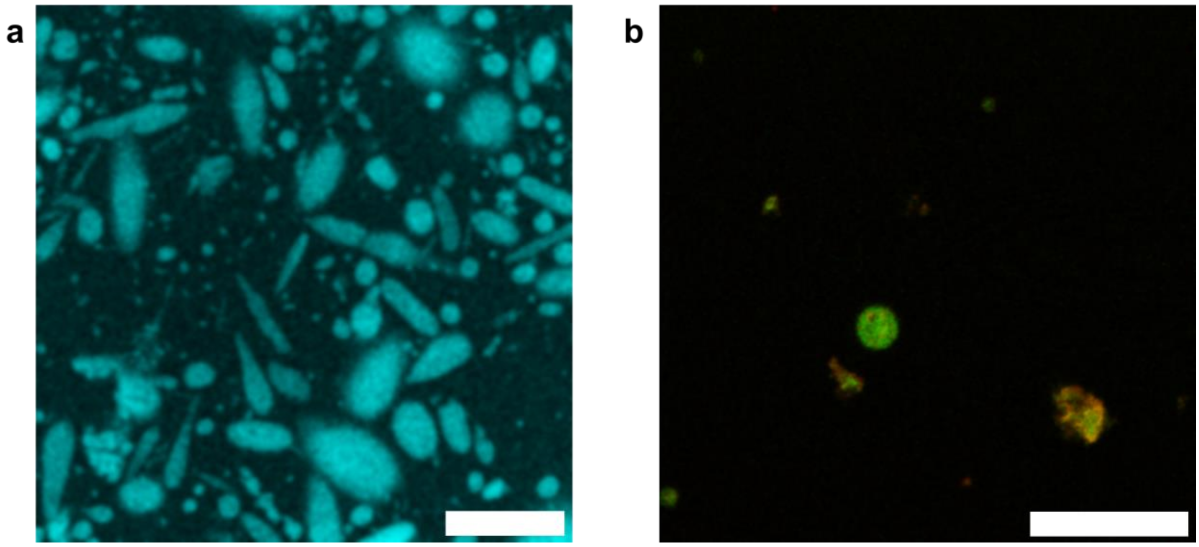

Figure S23. Overview CLSM micrographs **of** GUVs during enzymatic activity. (a) Resorufin produced by Mb. (b) Fluorescein (green) produced by β-gal (red: Cy5-PEG$_{3.5k}$-cholesterol). Scalebars for both images: 10 µm. Figure a has no membrane staining.

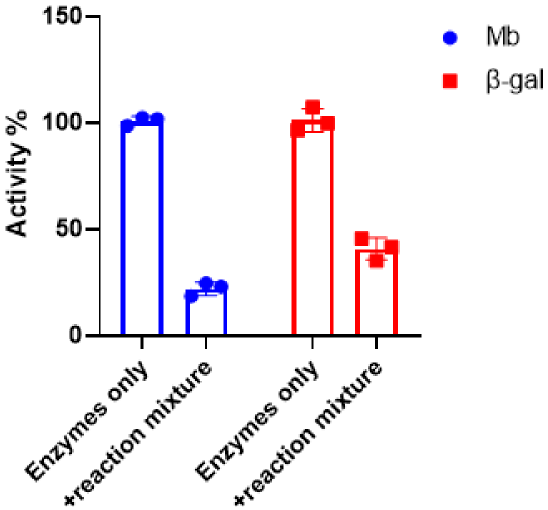

Figure S24. Activity of Mb and β-gal before and after incubation in the bioPISA reaction mixture, Mean ±SD, n = 3 replicates.

ACTIN POLYMERIZATION

The resulting structures were similar to intracellular phase transitions driven by intrinsically disordered proteins, rather than previously seen filaments (**Figure S25**).[13] These structures could not be observed in absence of Mg^{2+}(**Figure S26**), nor with only the added cation, without actin (**Figure S27**), confirming that only the interplay between polymerized actin and polymer membrane yielded these unique structures.

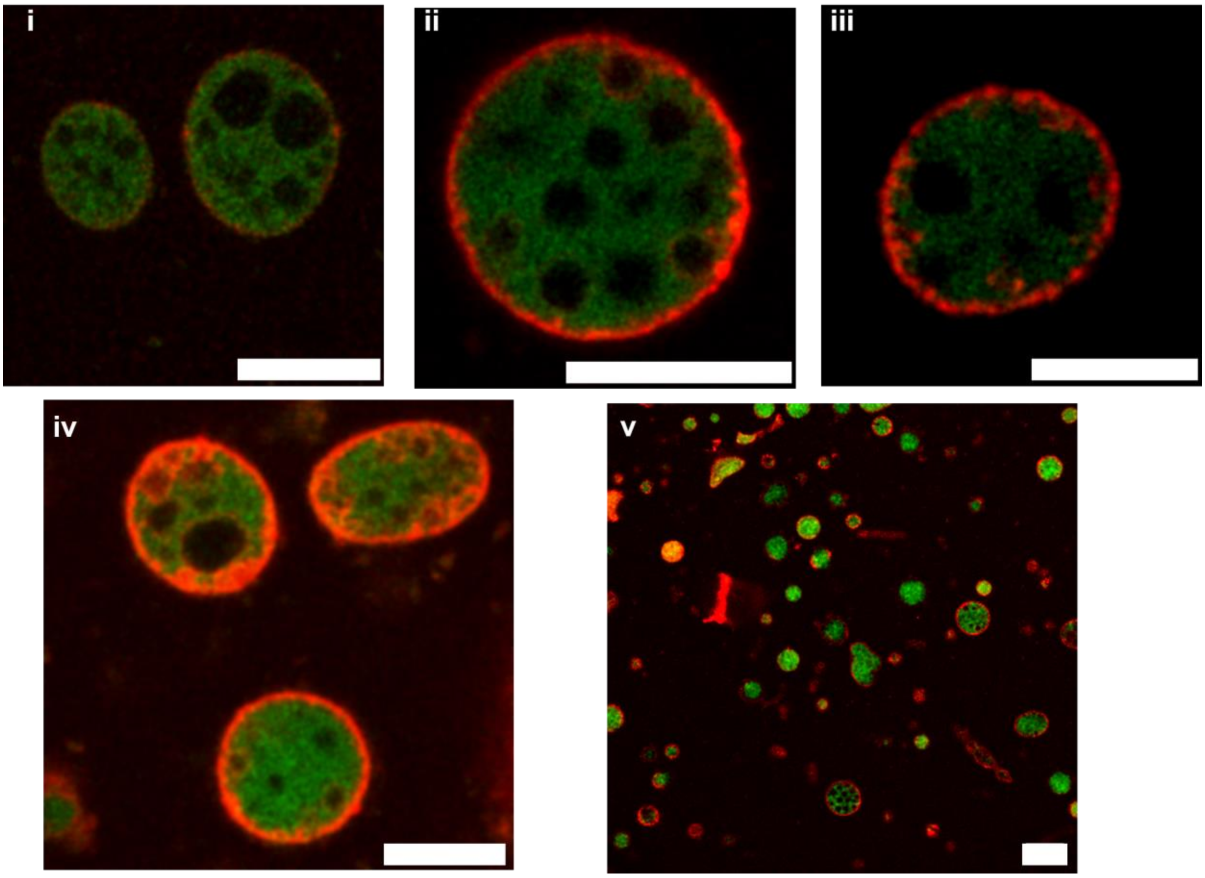

Figure S25. i-v) Several CLSM micrographs of bioPISA-derived GUVs after the formation of actin condensates (actin/actin-Atto488/filamin: 1/0.25/0.01 weight ratio; green: actin-Atto488, red: Cy5-PEG$_{3.5k}$-cholesterol; the cholesterol dye could only label some of the inner compartments)**.** Scalebars for all images: 10 µm.

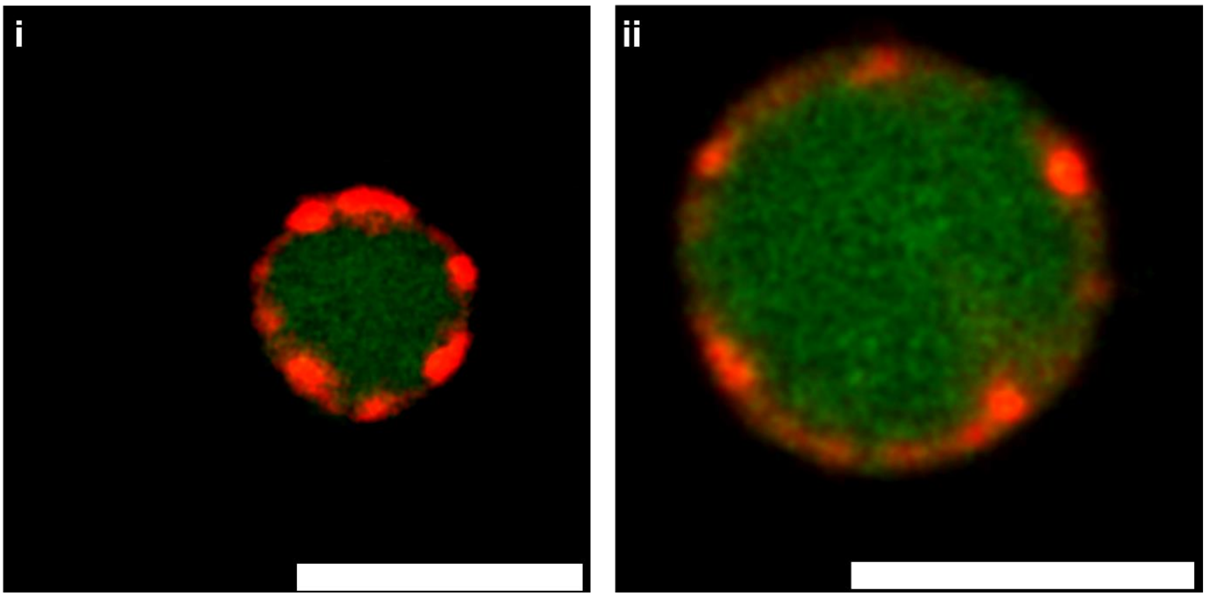

Figure S26. i-ii) Several CLSM micrographs of bioPISA-derived GUVs before the formation of actin condensates (actin/actin-Atto488/filamin: 1/0.25/0.01 weight ratio; green: actin-Atto488, red: Cy5-PEG$_{3.5k}$-cholesterol) Scalebars for both images: 5 µm.

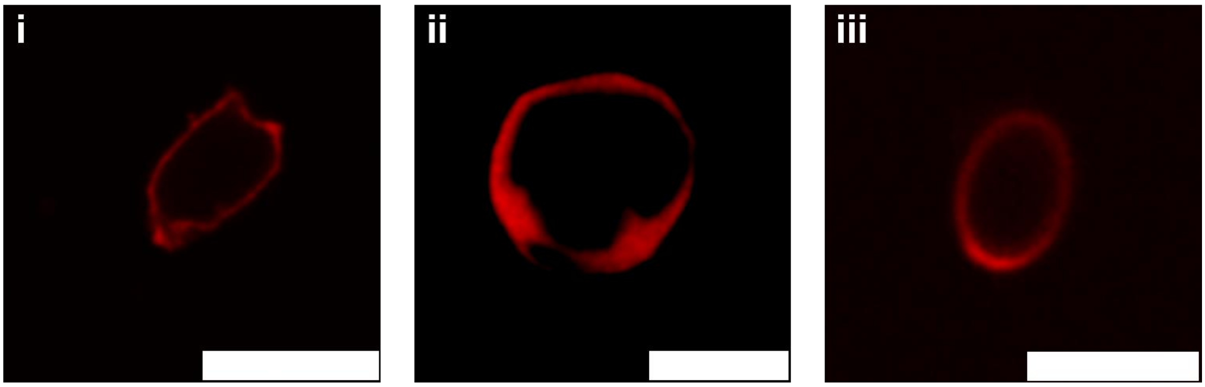

Figure S27. i-iii) Several CLSM micrographs of bioPISA-derived empty GUVs after addition of MgCl2. Red: Cy5-$PEG_{3.5k}$-cholesterol. The higher magnesium ion concentration appears to produce some deformations in the GUVs, but no subcompartment can be observed. Scalebars for all images: 10 µm.

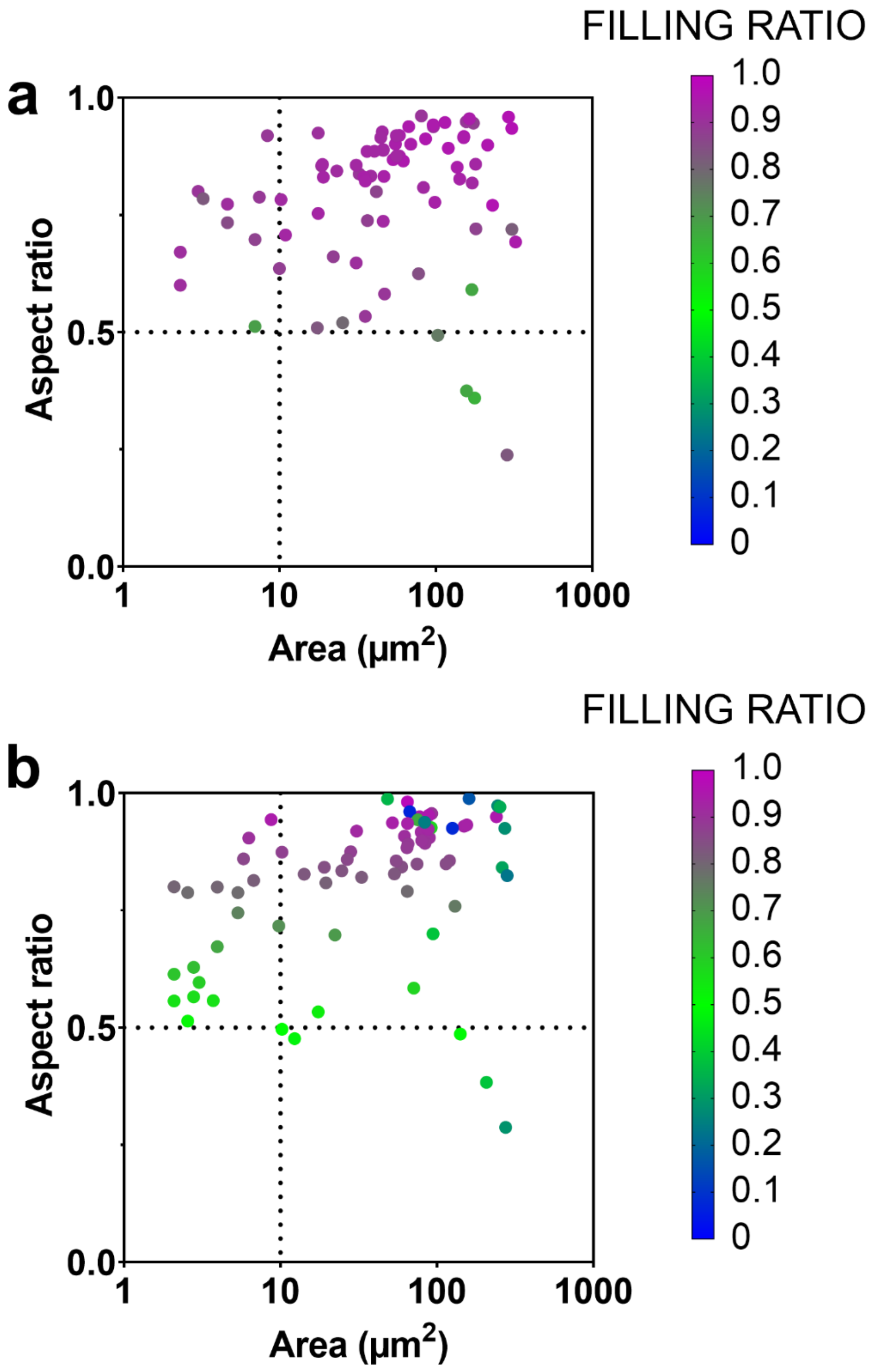

Figure S28. Scatter plots of the relation between size of GUVs encapsulating actin, their aspect ratio and their filling ratio (colour gradient), indicating a decrease in filling ratio, and thus a morphological reorganization upon actin polymerization. a) GUVs with actin in medium without $MgCl_2$ (n = 79 GUVs). b) GUVS with actin in MgCl2 (n = 73 GUVs). The additional line at 10 μm^2 indicates an arbitrary delimitation between smaller and larger GUVs; the line at aspect ratio 0.5 indicates an arbitrary delimitation between rounder and more elongated shapes. See Methods for the operational definition and measurement of filling ratio.

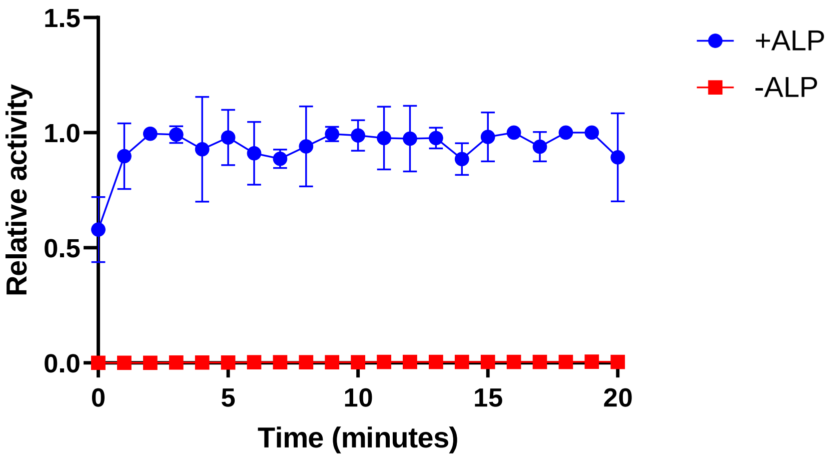

Figure S29. Activity of encapsulated ALP in bioPISA GUVs hydrolysing p-nitrophenyl phosphate (pNPP) to produce the phosphate-less coloured species pNP, showing that the enzyme is still active in the GUVs. All values displayed as mean ±SD, n=3 replicates for all experiments (some error bars are smaller than the symbols).

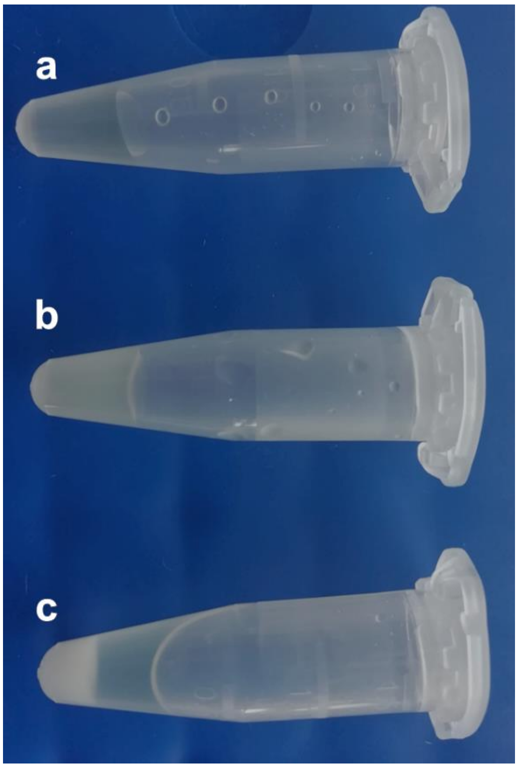

Figure S30. Co-precipitation of calcium phosphate and ALP in GUVs. a) CaGP, no GUVs. b) GUVs, no CaGP. c) GUVs + CaGP, leaving a clear supernatant in the last sample.

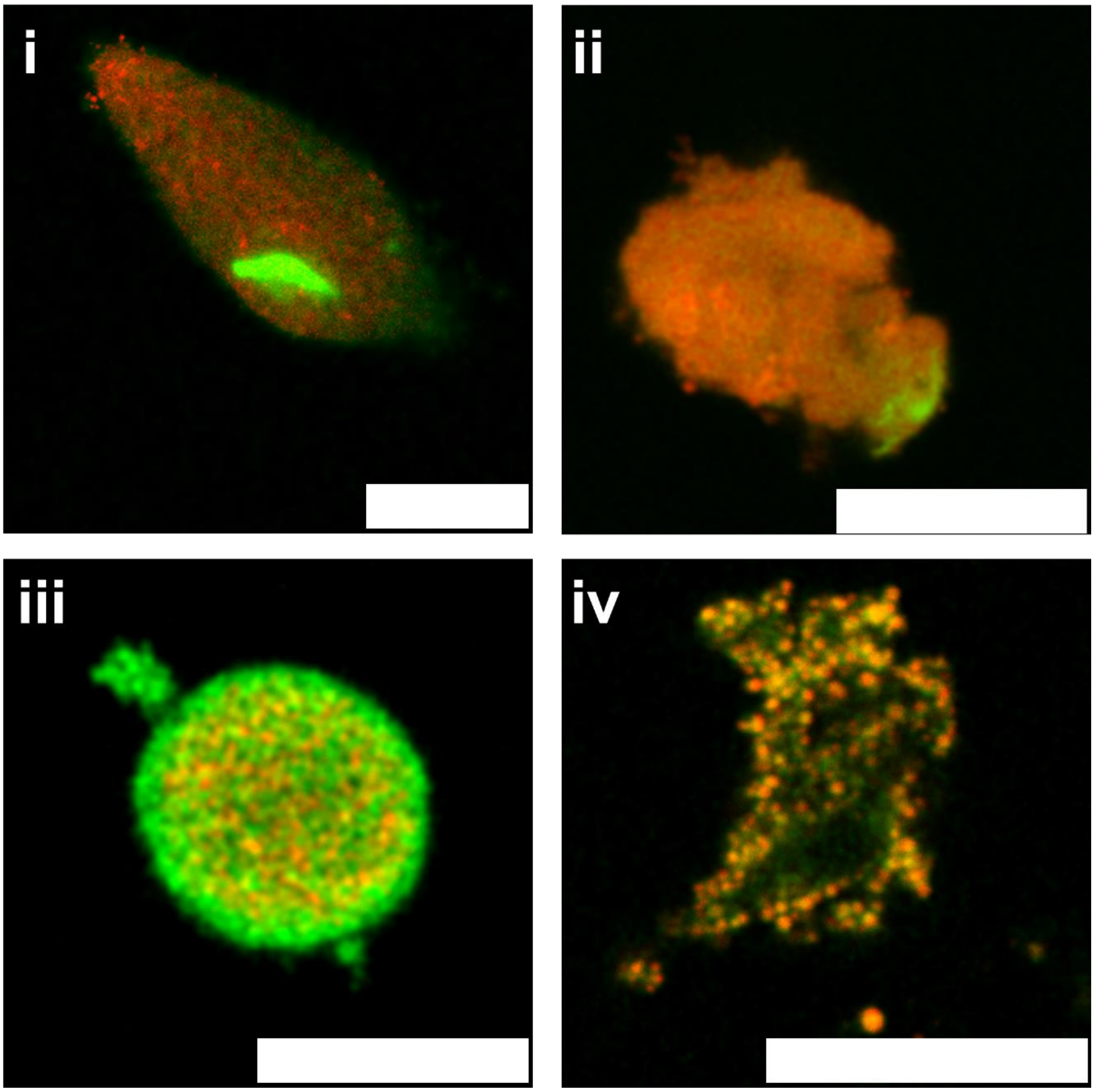

Figure S31. i – iv) Several CLSM micrographs of GUVs after the biomineralization of CaGP by ALP. Green: calcium phosphate-adsorbed fluorescein. Red: Cy5-PEG$_{3.5k}$-cholesterol. Scalebars for all images: 10 µm.

a

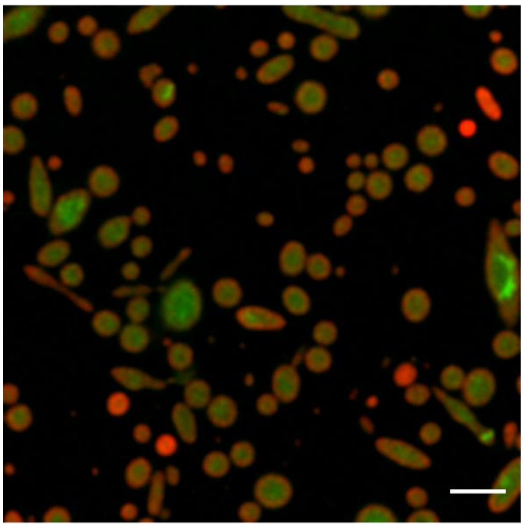

b

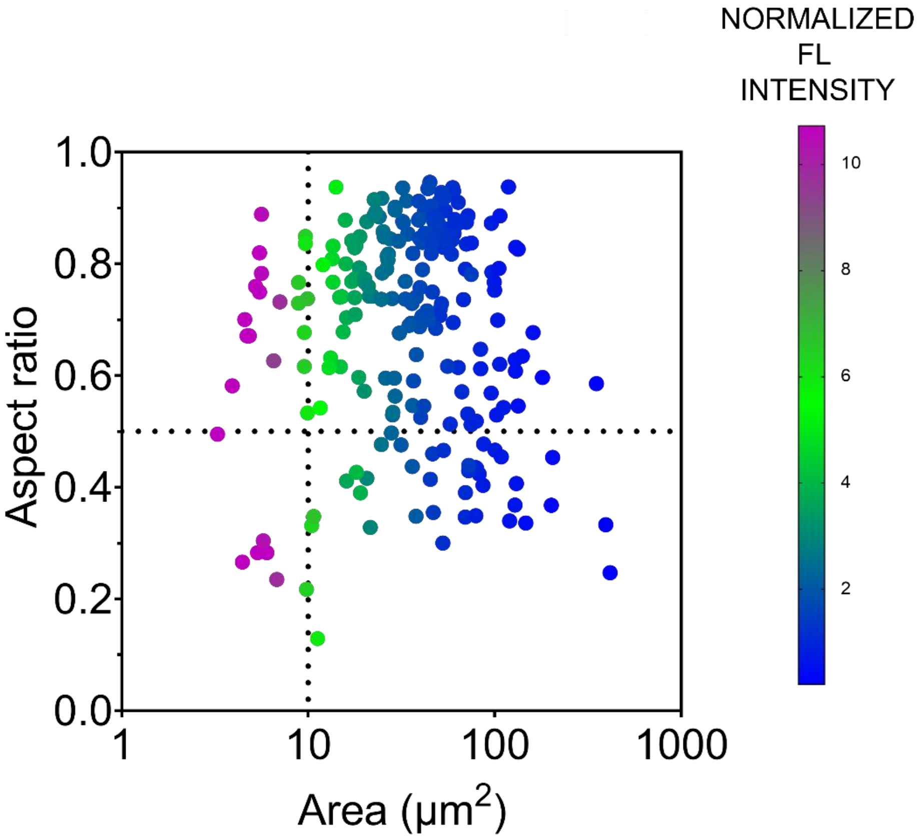

Figure S32. a) Representative overview CLSM micrograph of GUVs encapsulating cell lysate after expression of mClover (green: mClover, red: Cy5-PEG$_{3.5k}$-cholesterol). Some protein aggregates appear to be observed in some GUVs. Scalebar: 10 µm. b) Scatter plot of the relation between size of GUVs, their aspect ratio and their area-normalized fluorescence intensity (colour gradient). N = 230, maximum gradient value defined as 95% percentile.

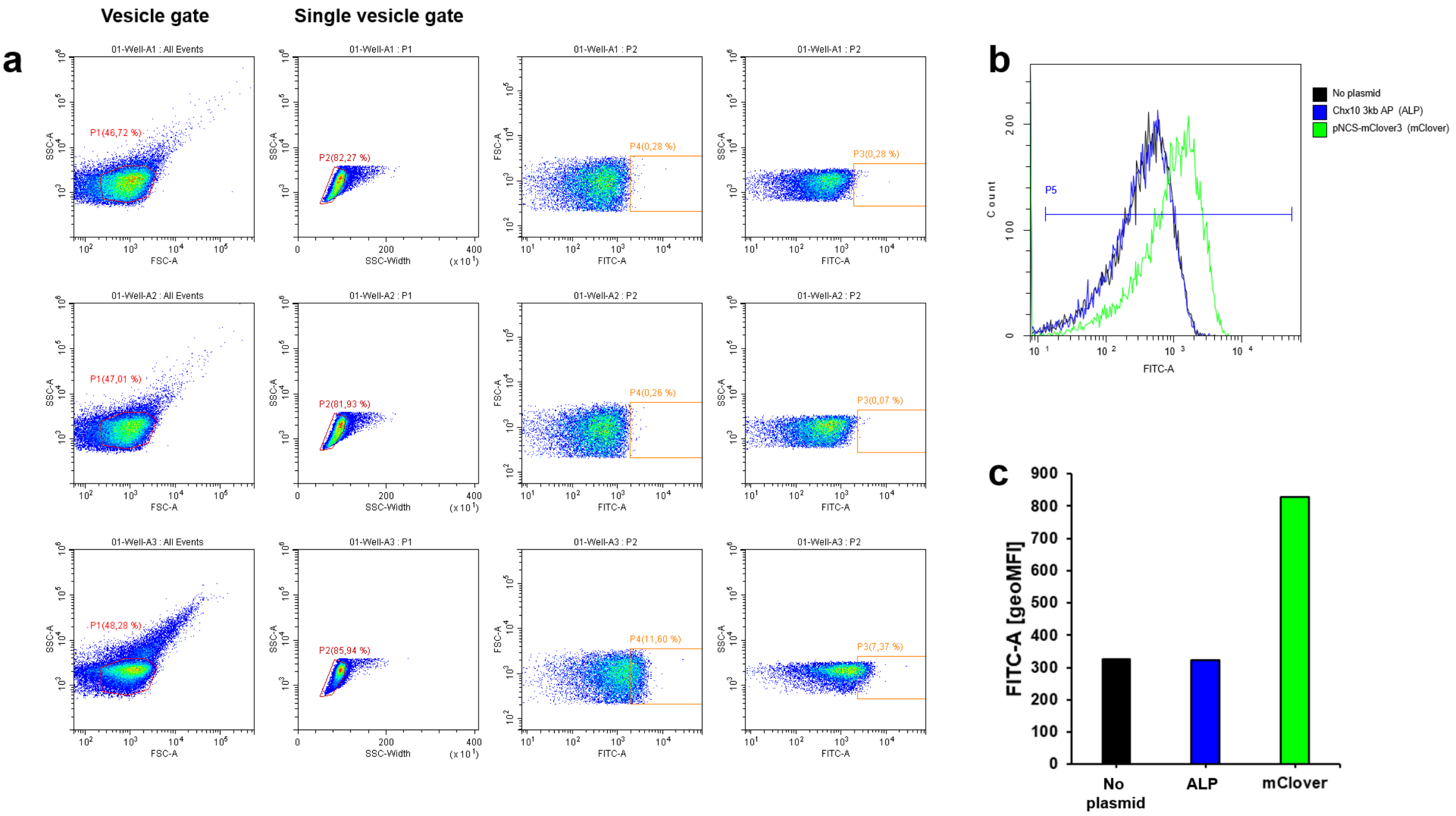

Figure S33. Flow cytometry fluorescence distributions for GUVs (FITC channel). a) Gating for well A1 (GUVs without plasmid), A2 (GUVs with ALP plasmid) and A3 (GUVs with mClover plasmid). b) Histograms for wells A1 to A3. P5: region for FITC fluorescence integration. c) Geometric mean of fluorescence intensity in FITC channel (P5).

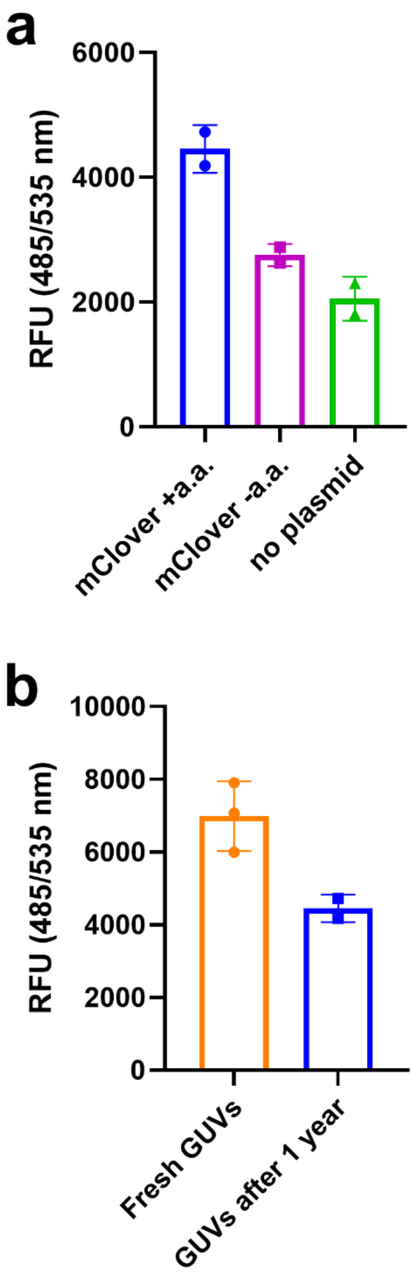

Figure S34. Stability of TX/TL expression system in GUVs. a) Fluorescence of mClover-expressing GUVs after 3 h. b) Comparison of activity (as fluorescence intensity) after 3 h for freshly-prepared GUVs and GUVs stored at 4°C for one year. All values displayed as mean ±SD, n = 3 for fresh GUVs, n = 2 replicates (no SD).

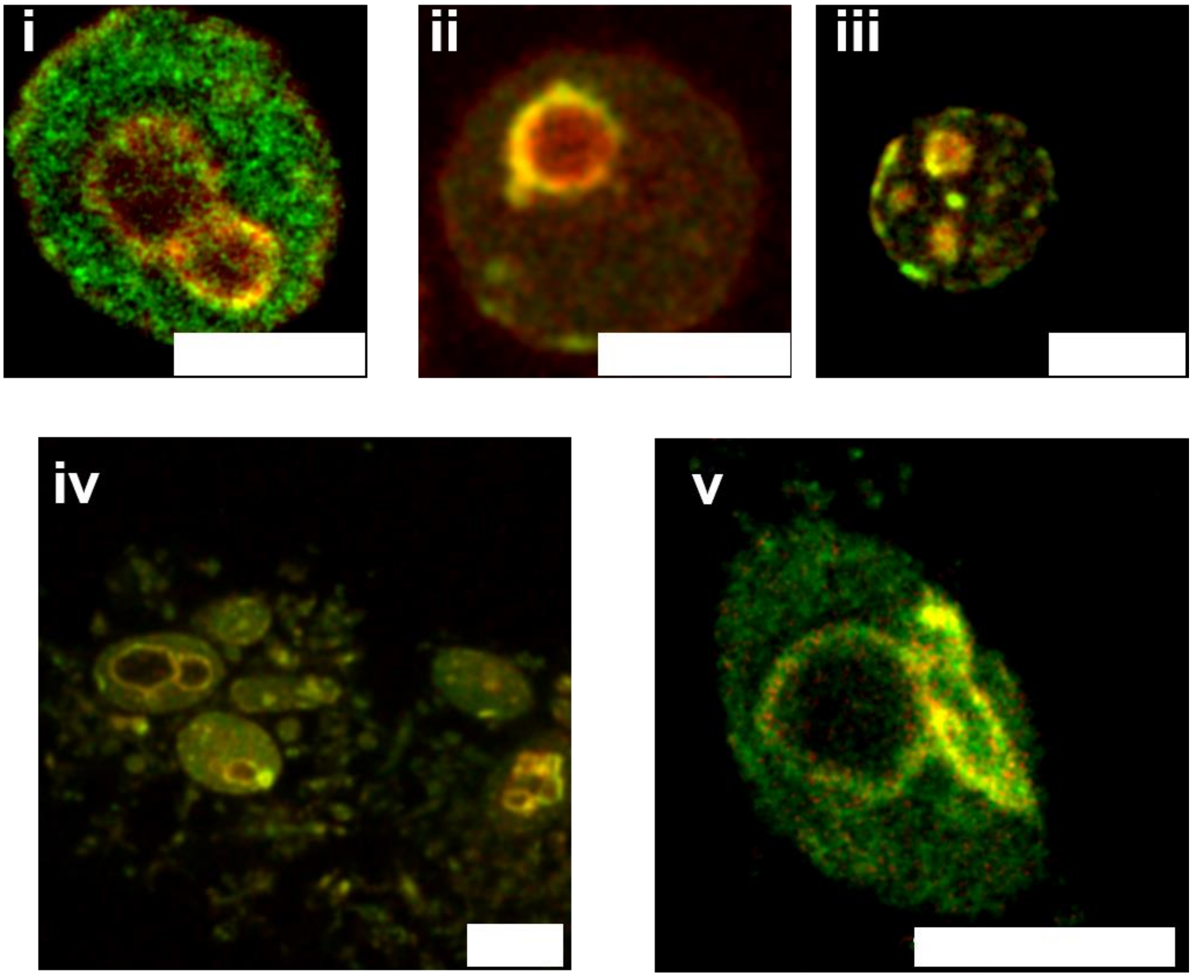

Figure S35. i –v) CLSM micrographs of GUVs with expressed actin + phalloidin-Atto565 (green: phalloidin-Atto565, red: Cy5-PEG$_{3.5k}$-cholesterol)**.** Scalebars for all images: 10 µm**.** Actin expression levels likely vary from GUV to GUV, and thus the morphologies it produces. In most cases, the inner compartments include stained membrane.

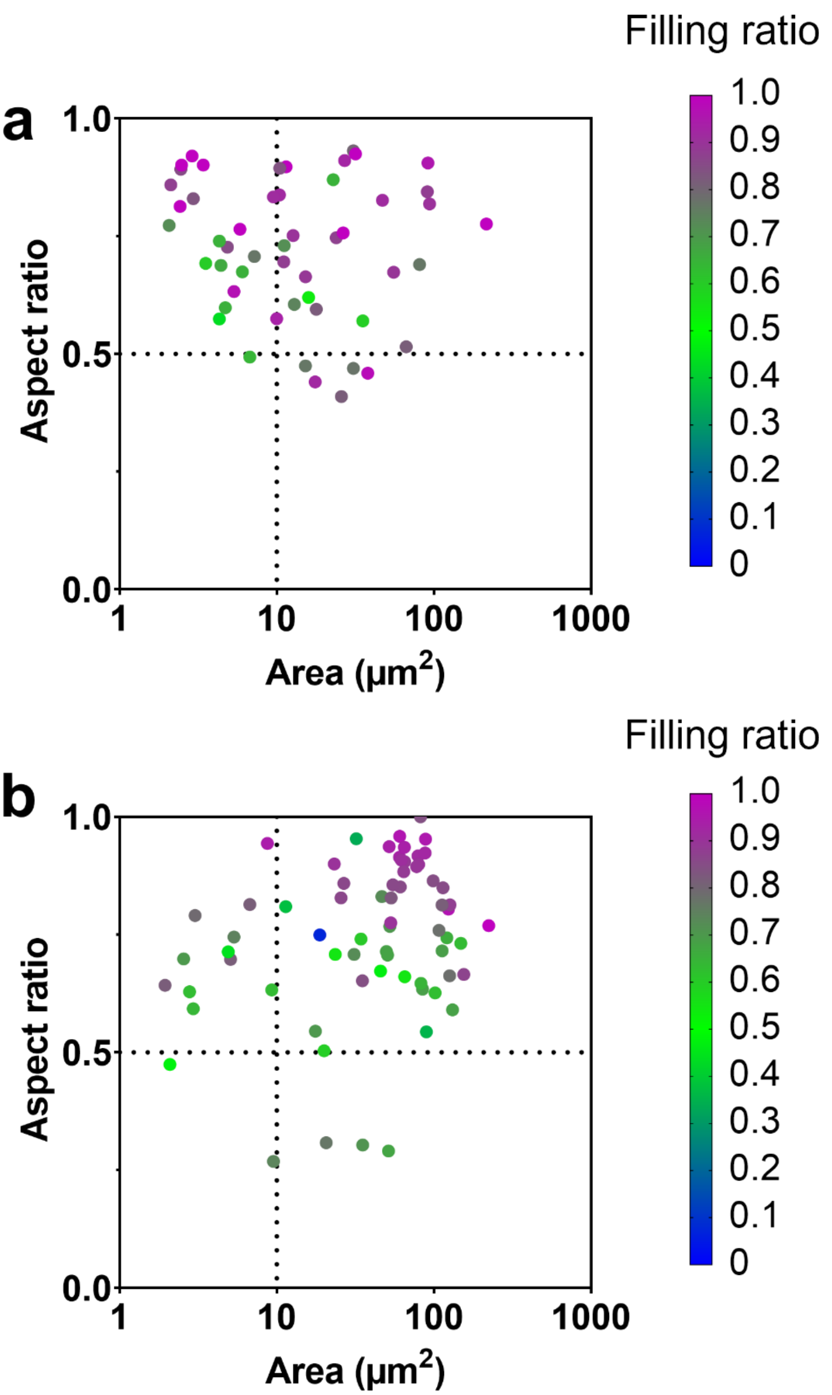

Figure S36. Scatter plots of the relation between size of GUVs expressing actin, their aspect ratio and their filling ratio (colour gradient), indicating a decrease in filling ratio, and thus a morphological reorganization. a) GUVs with actin in medium without MgCl2 (n = 51). b) GUVS with actin in $MgCl_2$ (n = 70). The additional line at 10 μm^2 indicates an arbitrary delimitation between smaller and larger GUVs; the line at aspect ratio 0.5 indicates an arbitrary delimitation between rounder and more elongated shapes. See Methods for the operational definition and measurement of filling ratio.

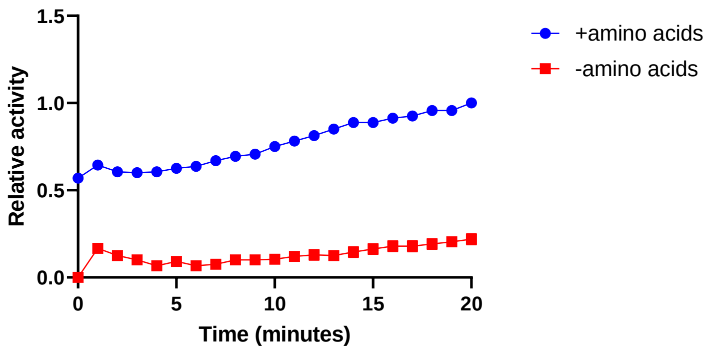

Figure S37. Activity of GUVs expressing ALP (decreased expression without supplemented amino acids) hydrolysing *p*-nitrophenyl phosphate (pNPP) to produce the phosphate-less coloured species pNP, plateauing earlier for GUVs + amino acids. All values displayed as mean ±SD (values too small to be displayed), n=3 replicates for all experiments.

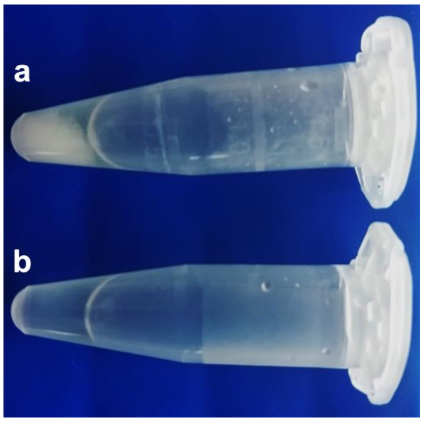

Figure S38. Co-precipitation of calcium phosphate and expressed ALP in GUVs. a) GUVs + expressed ALP + CaGP. b) GUVs + CaGP but no ALP expression.

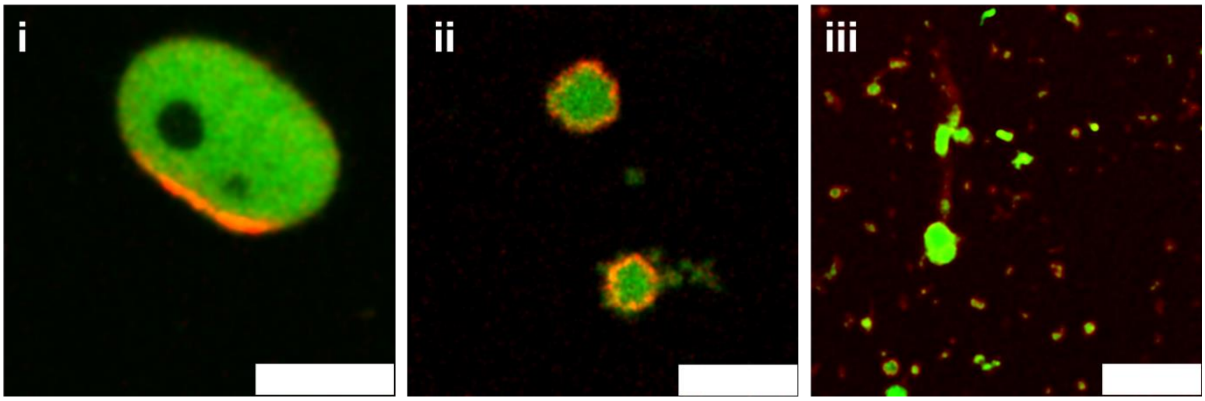

Figure S39. i-iii) Several CLSM micrographs of GUVs after the biomineralization of CaGP by expressed ALP. Green: calcium phosphate-adsorbed fluorescein. Red: Cy5-PEG$_{3.5k}$-cholesterol. Scalebars for all images: 10 µm. S39 iii is a micrograph in the same region of Figure 6g, lower focal plane.

1 Moreno-Echeverri, A. M. *et al.* Pitfalls in methods to study colocalization of nanoparticles in mouse macrophage lysosomes. *Journal of Nanobiotechnology* **20**, 464, doi:10.1186/s12951-022-01670-9 (2022).
2 Bajar, B. T. *et al.* Improving brightness and photostability of green and red fluorescent proteins for live cell imaging and FRET reporting. *Sci. Rep.* **6**, 20889, doi:10.1038/srep20889 (2016).
3 Rowan, S. & Cepko, C. L. A POU factor binding site upstream of the Chx10 homeobox gene is required for Chx10 expression in subsets of retinal progenitor cells and bipolar cells. *Dev. Biol.* **281**, 240-255, doi:10.1016/j.ydbio.2005.02.023 (2005).
4 de Graaf, A. J. *et al.* ATRP, subsequent azide substitution and 'click' chemistry: three reactions using one catalyst in one pot. *Chem. Commun.* **47**, 6972-6974, doi:10.1039/C1CC12224J (2011).
5 Khor, S. Y., Truong, N. P., Quinn, J. F., Whittaker, M. R. & Davis, T. P. Polymerization-Induced Self-Assembly: The Effect of End Group and Initiator Concentration on Morphology of Nanoparticles Prepared via RAFT Aqueous Emulsion Polymerization. *ACS Macro Lett.* **6**, 1013-1019, doi:10.1021/acsmacrolett.7b00583 (2017).
6 Teodorescu, M. & Matyjaszewski, K. Atom Transfer Radical Polymerization of (Meth)acrylamides. *Macromolecules* **32**, 4826-4831, doi:10.1021/ma990175x (1999).
7 D'hooge, D. R. *et al.* Assessment of end-group functionality in atom transfer radical polymerization of N-isopropylacrylamide. *Eur. Polym. J.* **49**, 2344-2355, doi:https://doi.org/10.1016/j.eurpolymj.2013.05.019 (2013).
8 Ribelli, T. G., Lorandi, F., Fantin, M. & Matyjaszewski, K. Atom Transfer Radical Polymerization: Billion Times More Active Catalysts and New Initiation Systems. *Macromol. Rapid Commun.* **40**, 1800616, doi:https://doi.org/10.1002/marc.201800616 (2019).
9 Connell, L. S., Jones, J. R. & Weaver, J. V. M. Transesterification of functional methacrylate monomers during alcoholic copper-catalyzed atom transfer radical polymerization: formation of compositional and architectural side products. *Polymer Chemistry* **3**, 2735-2738, doi:10.1039/C2PY20280H (2012).
10 Save, M., Weaver, J. V. M., Armes, S. P. & McKenna, P. Atom Transfer Radical Polymerization of Hydroxy-Functional Methacrylates at Ambient Temperature: Comparison of Glycerol Monomethacrylate with 2-Hydroxypropyl Methacrylate. *Macromolecules* **35**, 1152-1159, doi:10.1021/ma011541r (2002).
11 Wang, G. *et al.* Polymerization-Induced Self-Assembly (PISA) Using ICAR ATRP at Low Catalyst Concentration. *Macromolecules* **49**, 8605-8615, doi:10.1021/acs.macromol.6b01966 (2016).
12 Shetty, S. C. *et al.* Directed Signaling Cascades in Monodisperse Artificial Eukaryotic Cells. *ACS Nano* **15**, 15656-15666, doi:10.1021/acsnano.1c04219 (2021).
13 Shin, Y. *et al.* Spatiotemporal Control of Intracellular Phase Transitions Using Light-Activated optoDroplets. *Cell* **168**, 159-171.e114, doi:10.1016/j.cell.2016.11.054 (2017).
